# Biochemical and crystallographic studies of l,d-transpeptidase 2 from *Mycobacterium tuberculosis* with its natural monomer substrate

**DOI:** 10.1038/s42003-024-06785-3

**Published:** 2024-09-18

**Authors:** Mariska de Munnik, Pauline A. Lang, Karina Calvopiña, Patrick Rabe, Jürgen Brem, Christopher J. Schofield

**Affiliations:** 1 Chemistry Research Laboratory, Department of Chemistry and the Ineos Oxford Institute of Antimicrobial Research, University of Oxford, Oxford, UK; 2 Present Address: Enzymology and Applied Biocatalysis Research Center, Faculty of Chemistry and Chemical Engineering, Babes-Bolyai University, Cluj-Napoca, Romania

**Keywords:** Structural biology, Enzyme mechanisms

## Abstract

The essential l,d-transpeptidase of *Mycobacterium tuberculosis* (Ldt_Mt2_) catalyses the formation of 3$\to$3 cross-links in cell wall peptidoglycan and is a target for development of antituberculosis therapeutics. Efforts to inhibit Ldt_Mt2_ have been hampered by lack of knowledge of how it binds its substrate. To address this gap, we optimised the isolation of natural disaccharide tetrapeptide monomers from the *Corynebacterium jeikeium* bacterial cell wall through overproduction of the peptidoglycan sacculus. The tetrapeptides were used in binding / turnover assays and biophysical studies on Ldt_Mt2._ We determined a crystal structure of wild-type Ldt_Mt2_ reacted with its natural substrate, the tetrapeptide monomer of the peptidoglycan layer. This structure shows formation of a thioester linking the catalytic cysteine and the donor substrate, reflecting an intermediate in the transpeptidase reaction; it informs on the mode of entrance of the donor substrate into the Ldt_Mt2_ active site. The results will be useful in design of Ldt_Mt2_ inhibitors, including those based on substrate binding interactions, a strategy successfully employed for other nucleophilic cysteine enzymes.

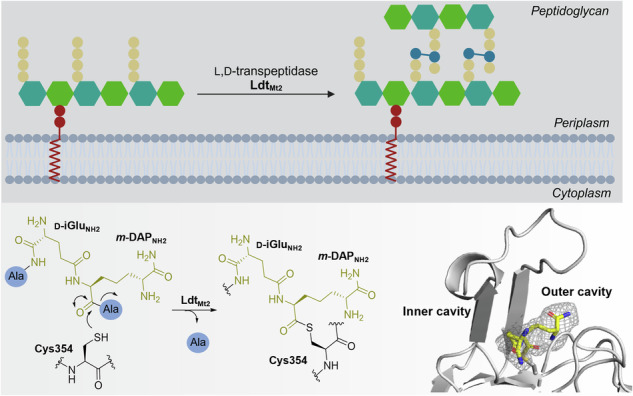

## Abstract (web summary)

Studies of the reaction between an essential L,D-transpeptidase of *Mycobacterium tuberculosis* and its natural substrate monomer inform on its binding mode; one crystal structure reflects an S-acyl intermediate in the transpeptidase reaction.

## Introduction

Tuberculosis (TB) imposes a major impact on global health and causes an estimated 1.6 million deaths per year^1^. There is a limited set of drugs for TB treatment; however, the prolonged treatment regimens required for efficacy and increasing resistance mean there is a clear need for new therapeutics targeting *Mycobacterium tuberculosis* (*Mtb*), the causative agent of TB^2,3^.

**Fig. 1:**
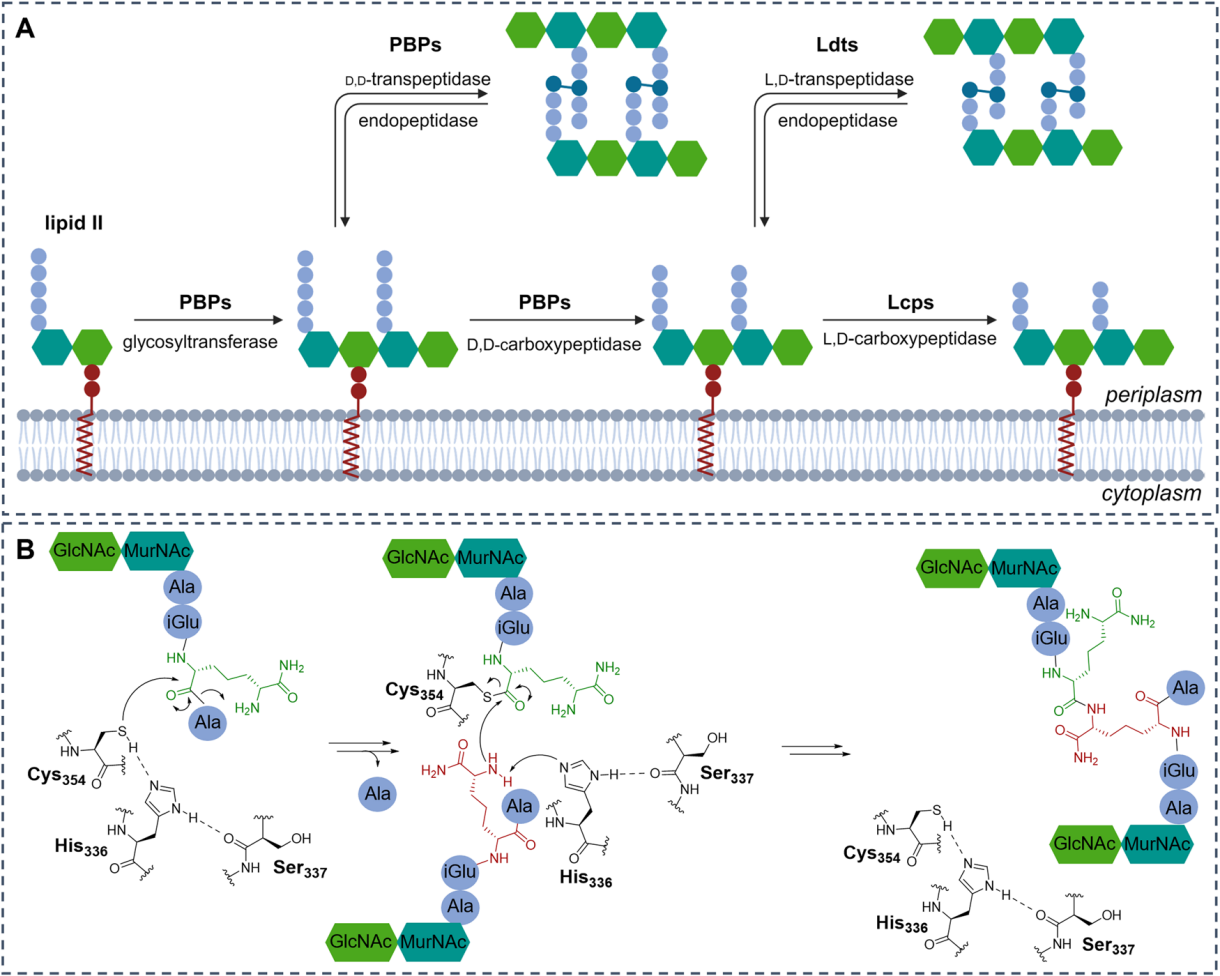
Roles of glycosyltransferases, transpeptidases and carboxypeptidases in the final stages of bacterial peptidoglycan synthesis. **A** Penicillin binding proteins (PBPs) with glycosyltransferase activity link lipid II to peptidoglycan, PBPs with carboxypeptidase activity cleave peptide strands from pentapeptides to give tetrapeptides and PBPs with transpeptidase activity form 4$\to$3 cross-links between pentapeptide strands. The l,d-transpeptidases (Ldts) form 3$\to$3 cross-links between tetrapeptide strands. Endopeptidases reverse the transpeptidase activity, cleaving the peptide bonds between cross-links. Lcps cleave peptide strands from tetrapeptides to tripeptides. The figure was created using BioRender.com. GlcNAc is in green, MurNAc is in teal, amino acids are in light blue, cross-linked amino acids are in dark blue. **B** Mechanism for the cross-linking reaction between the disaccharide tetrapeptide monomers catalysed by Ldt_Mt2_ (adapted from de Munnik et al.^26^).

Disruption of cell wall biosynthesis is arguably the most effective current approach for treatment of bacterial infections, with the cell wall biosynthesis targeting β-lactams being amongst the most successful of antibacterial drug classes^4^. β-Lactams are efficient and normally safe inhibitors of the penicillin binding proteins (PBPs), a class of nucleophilic serine enzymes that are involved in several of the final steps of peptidoglycan synthesis; PBPs include d,d-transpeptidases (some with bifunctional glycosyltransferase activity), d,d-carboxypeptidases, and endopeptidases (Fig. 1A)^5^. To date, the application of β-lactams for the treatment of TB has been limited, reflecting challenges including β-lactamase mediated resistance^6^. A further issue with β-lactam based TB treatment is that in addition to the nucleophilic serine PBPs, nucleophilic cysteine enzymes play a key role in *Mtb* cell wall biosynthesis^7^.

Whereas the d,d-transpeptidases catalyse the formation of 4$\to$3 cross-links between peptidoglycan pentapeptide chains, the functionally related l,d-transpeptidases (Ldts) catalyse the formation of 3$\to$3 cross-links between tetrapeptide chains (Fig. 1)^8,9^. Despite being well conserved between bacterial species^10^, the low abundancy of 3$\to$3 cross-links is proposed to render Ldts redundant in most bacteria, at least in controlled laboratory environments^11^. However, *Mtb* displays up to 80% of 3$\to$3 cross-links during the stationary phase^7^. Hence, the Ldts have been identified as attractive targets for the development of anti-TB treatment^12^. In particular Ldt_Mt2_, one of the five Ldts of *Mtb*, is recognised as being important for virulence^13–15^.

Whereas the d,d-carboxypeptidases and d,d-transpeptidases are nucleophilic serine enzymes (Fig. S1)^16,17^, the Ldts employ a catalytic triad involving the sidechains of a nucleophilic cysteine and a histidine residue, and the backbone carbonyl of a third residue in a characteristic Hxx_14–17_(S/T)HGCZN motif, in which Z represents a hydrophobic residue^18^. The Ldts, which do not manifest structural homology with the PBPs and likely originate from a different evolutionary precursor^18^, share a common YkuD fold (named after the first identified member of this class), distinguished by a β-sandwich with two mixed β-sheets and one helix, and a set of four loops (Fig. S1)^18–20^. One of these loops borders the Ldt_Mt2_ active site^19^, creating two entrances towards the nucleophilic cysteine: the inner cavity and the outer cavity (Fig. S1)^19^.

**Fig. 2:**
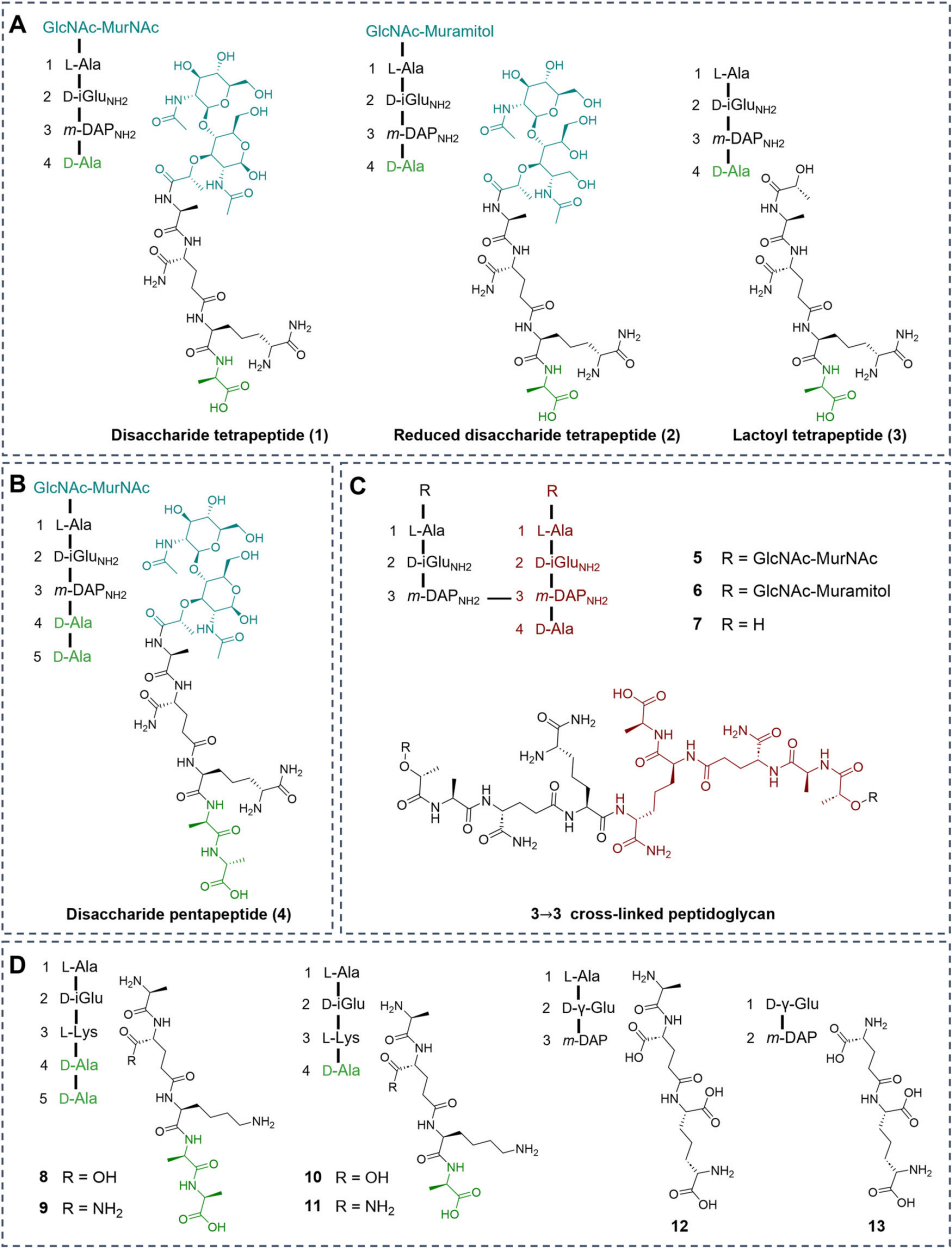
Structures of peptidoglycan fragments of *Mycobacterium tuberculosis.* **A** Structures of the tetrapeptide substrates of Ldt_Mt2_ **1**-**3**, obtained through isolation from the cell wall of *C. jeikeium*. **B** Structure of the pentapeptide monomer of peptidoglycan from *Mtb* and *C. jeikeium* **4**, obtained through isolation from the cell wall of *C. jeikeium*. **C** The structures of the products of transpeptidase activity of Ldt_Mt2_ **5**-**7**, obtained through cross-linking of **1,** **2** and **3**, respectively. **D** Peptidoglycan fragments **8**-**13** that do not act as substrates for Ldt_Mt2_, were obtained through solid-phase peptide synthesis (**9** and **11**) or commercial sources (**8,** **10,** **12** and **13**). GlcNAc stands for N-acetylglucosamine, MurNAc stands for *N*-acetylmuramic acid.

The established substrate for Ldt_Mt2_ is the disaccharide tetrapeptide monomer **1** (GlcNAc-MurNAc-l-Ala-d-iGlu_NH2_-*m-*DAP_NH2_-d-Ala; Fig. 2A)^7,13^. The formation of 3$\to$3 crosslinks is proposed to occur through covalent reaction of the amide bond between the 3^rd^ and 4^th^ residue with the nucleophilic cysteine of Ldt_Mt2_, i.e., Cys354, to give an acyl-enzyme complex^21^. The 3^rd^ residue of a second strand of the tetrapeptide chain then reacts with the thioester intermediate to give the 3$\to$3 crosslinked product (Fig. 1B)^22^.

Though traditionally viewed as PBP inhibitors, selected β-lactams also inhibit the Ldts. In particular, carbapenems have been shown to inhibit Ldts^23–25^, and recent work has shown the potential of cephalosporins to inhibit Ldt_Mt2_^26^. Several other classes of covalently reacting electrophilic compounds, e.g., ebselen, nitriles and acrylamides, have also been reported to inhibit Ldt_Mt2_^26,27^. Structure-based design of Ldt inhibitors has, however, been limited by a lack of detailed knowledge of enzyme-substrate interactions.

Studies of Ldt_Mt2_ in complex with its substrate or acyl-enzyme intermediate have been hindered by the restricted availability of **1**. Reported studies of the Ldt_Mt2_-substrate complex have thus far relied on unnatural substrate analogues, e.g., a crystal structure of the peptidoglycan fragment d-γ-Glu-*m*-DAP bound to the Ldt_Mt2_ active site^22^. Efforts to chemically synthesise substrate **3** (lactoyl-l-Ala-d-iGlu_NH2_-*m-*DAP_NH2_-d-Ala) have yielded only low amounts of material^28^. The peptidoglycan substrates **1,** **2** (GlcNAc-Muramitol-l-Ala-d-iGlu_NH2_-*m-*DAP_NH2_-d-Ala), and **3** can, however, be obtained through isolation from the cell well of *Corynebacterium jeikeium*, which shares an identical peptidoglycan monomer structure with *Mtb*^7,13,29^. Using a variation of this method, we obtained **1**-**3** from the cell wall of *C. jeikeium* in significantly improved yields, enabling detailed studies on their interaction with Ldt_Mt2_. Kinetic and binding studies suggest that isolated Ldt_Mt2_ has a low affinity for the isolated tetrapeptide peptidoglycan monomer. X-ray crystallographic studies of Ldt_Mt2_ and substrate **3** yielded a structure of the proposed thioester intermediate of the transpeptidase reaction, which suggests the donor substrate enters through the inner cavity, but subsequently interacts with both the inner and outer cavities.

## Results

### Isolation of the tetrapeptide monomer

Initially, we isolated the reduced disaccharide tetrapeptide monomer **2** from the cell wall of *C. jeikeium* using reported methods^29,30^. *C. jeikeium* cells were lysed via high-pressure homogenesis, boiled in 8% sodium dodecyl sulphate, then treated with trypsin, pronase, mutanolysin, and lysozyme to yield soluble peptidoglycan monomers (Figure S2). MurNAc moieties in peptidoglycan monomers were then reduced to muramitol using sodium borohydride, to improve resolution during HPLC separation (Fig. S3A)^30^. This method was scaled to a dried cell mass of 94.6 g *C. jeikeium* from 21.6 L of culture, which yielded ∼4.5 mg of **2** (Fig. S4A). Aiming to improve the yield, we attempted to bypass the reduction of MurNAc in **1**. Decreased separation of peaks was observed by HPLC (Fig. S3B); however, highly purified (>95%) disaccharide-tetrapeptide **1** (Fig. S4B) was obtained (∼3.6 mg from 61.3 g dried cell mass).

As described^29^, we observed that the distribution between tetrapeptide monomer **1** and pentapeptide monomer **4** favours **1** in crude samples. Addition of ampicillin in apparently subinhibitory concentrations (2 μg/mL)^29^ increased the abundance of **4** (Fig. S3C), likely due to ampicillin-mediated inhibition of d,d-carboxypeptidase and d,d-transpeptidase activities, leading to accumulation of their substrate (**4**)^5,17^. Subsequent incubation of isolated **4** with the purified recombinant *Escherichia coli* d,d-carboxypeptidase DacA gave **1** (Fig. S5). This procedure substantially (∼10 fold) improved the overall yield of **1** (∼20.5 mg from 55.9 g dried cell mass). The disaccharide moiety of **1** was cleaved by reaction with ammonium hydroxide^29,30^, giving lactoyl-tetrapeptide **3** (Fig. S6). The identities and purities of **1,** **2** and **3** were confirmed by liquid chromatography mass spectrometry (LC-MS; Fig. S4) and ^1^H and ^13^C NMR characterisation (Figs. S7 and 8).

### Studies of Ldt_Mt2_ activity with peptidoglycan monomers

We assessed Ldt_Mt2_ catalysis via LC-MS analysis of reaction mixtures of recombinant Ldt_Mt2_ with the three variants of l-Ala-d-iGlu_NH2_-*m-*DAP_NH2_-d-Ala: the peptide containing GlcNAc-MurNAc **1**, the peptide containing GlcNAc-Muramitol **2**, and the lactoyl peptide **3**. Incubation of Ldt_Mt2_ (5 µM) with **1,** **2**, or **3** (100 µM) led to formation of products **5,** **6** and **7**, as evidenced by the observation of species with masses of 1786 Da, 1790 Da, and 974 Da, respectively, (Fig. S9), as reported for **2** and **3**^13,28^.

Next, we assessed the structural requirements of the tetrapeptide residues that enable reaction with Ldt_Mt2_. A previous study identified the importance of sidechain amidation of both the d-iGlu and *m-*DAP residues for turnover^28^. To further investigate the structural requirements for Ldt_Mt2_ substrates, we incubated Ldt_Mt2_ with tetrapeptide **10** (l-Ala-d-iGlu-l-Lys-d-Ala), a peptidoglycan monomer that is often observed in Gram-positive bacteria^11^. We observed no evidence for turnover of **10**. The tetrapeptide **11** (l-Ala-d-iGlu_NH2_-l-Lys-d-Ala), which contains an amidated d-iGlu residue, but wherein *m-*DAP_NH2_ is replaced with l-Lys, was also not a substrate (over 24 h), evidencing the importance of the *m-*DAP_NH2_ residue in peptidoglycan fragments for turnover with isolated Ldt_Mt2_.

It is reported that the reduced pentapeptide monomer of *Mtb* (GlcNAc-muramitol-l-Ala-d-iGlu_NH2_-*m-*DAP_NH2_-d-Ala-d-Ala) is not an Ldt_Mt2_ substrate^13^. Consistent with this, incubation of Ldt_Mt2_ with the pentapeptide monomer of *Mtb* **4** (GlcNAc-MurNAc-l-Ala-d-iGlu_NH2_-*m-*DAP_NH2_-d-Ala-d-Ala), or pentapeptide **8** (l-Ala-d-iGlu-l-Lys-d-Ala-d-Ala), did not manifest evidence for reaction, further evidencing that pentapeptide monomers are not Ldt_Mt2_ substrates.

We were interested in assessing the transpeptidase activity of the active site cysteine to serine mutant of Ldt_Mt2_ (Ldt_Mt2_^C354S^), in part because previous reports have shown that in contrast to wild-type Ldt_Mt2_^13,21^, Ldt_Mt2_^C354S^ is unable to form a covalent complex with several penem and carbapenem derivatives^23,31^. When Ldt_Mt2_^C354S^ was incubated with **1,** **2,** **3** or **4**, no product formation was observed, suggesting the active site cysteine is essential for transpeptidase activity, and cannot be replaced by a potentially nucleophilic serine residue.

Differential scanning fluorometric thermal shift assays^32^ (Fig. S10 and Table S1) with a 100-fold excess (500 µM) of peptidoglycan fragments showed pentapeptide **4** induced a modest stabilising effect, as evidenced by a shift in the melting temperature (T_m_) of ≥2 °C. With a 200-fold excess (1 mM), stabilisation was observed with tetrapeptides **1,** **3** and **9**, and pentapeptides **4** and **8**, all manifesting a T_m_ shift of 2-3 °C; no significant shift in T_m_ was observed with **10** and **11**.

Protein-observed solid-phase extraction mass spectrometric (SPE-MS) studies were performed with Ldt_Mt2_ (1 µM) in the presence of **1** and **3** (100 µM). While transpeptidase activity was confirmed with **1** and **3**, as evidenced by observation of protein unbound species with masses of 1786 Da, and 974 Da, (correlating to **5** and **7**, respectively), no covalent adducts of Ldt_Mt2_ were observed, suggesting that in solution the lifetime of the covalent thioester (predicted mass increase of 849 Da in the case of **1** and 443 Da in the case of **3**, relative to unmodified Ldt_Mt2_) intermediate is short-lived (Fig. S11).

### Kinetics of the Ldt_Mt2_ transpeptidase reaction with peptidoglycan monomers

**Fig. 3:**
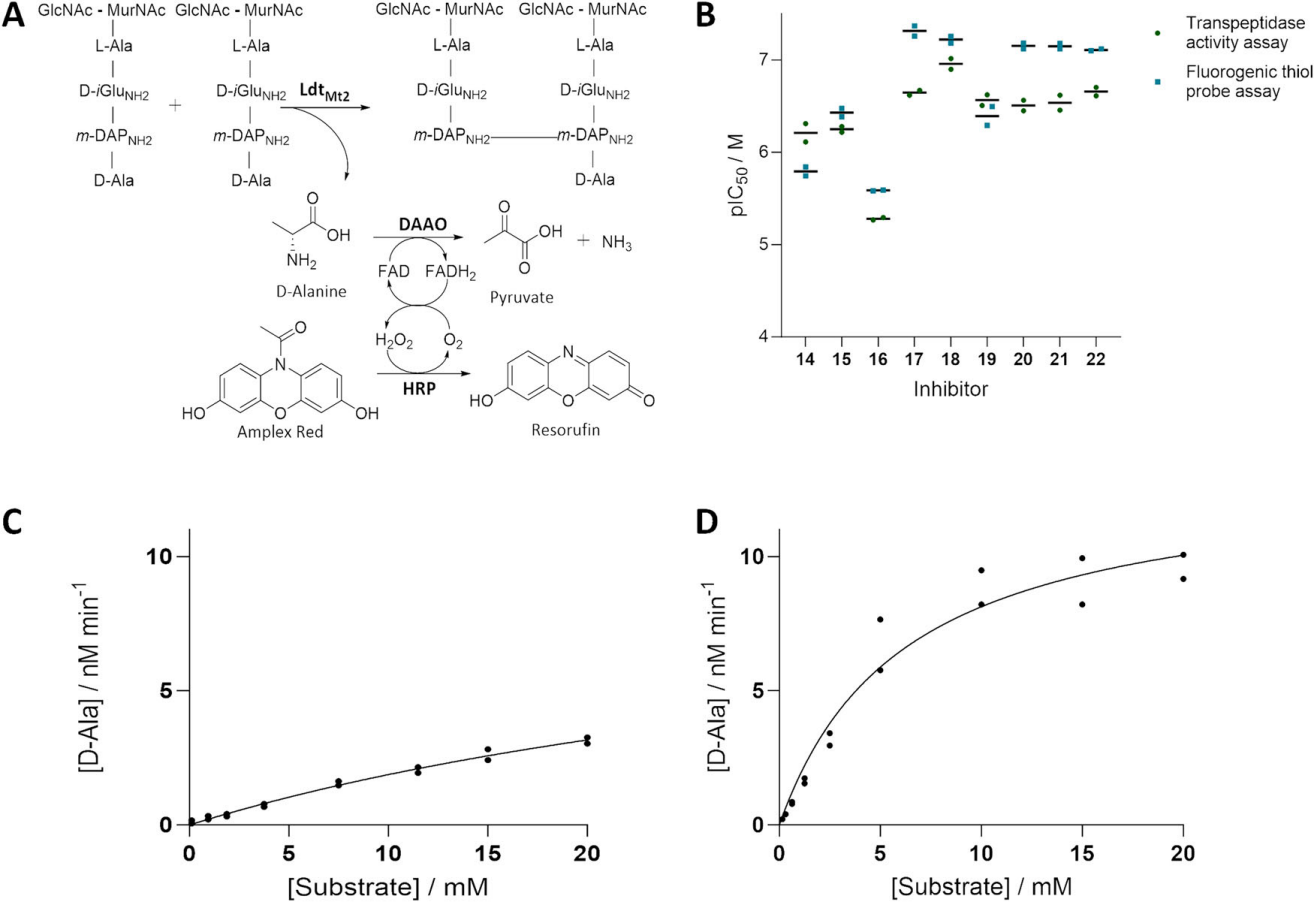
A fluorescence-based assay for Ldt_Mt2_ transpeptidase activity. **A** Principle of the Ldt_Mt2_ transpeptidase assay, employing d-amino acid oxidase (DAAO) and horseradish peroxidase (HRP) to report on the release of d-Ala. **B** Correlation between pIC_50_ values obtained with the transpeptidase activity assay and fluorogenic thiol probe assay^26^. Individual data points are shown. **C** Michaelis-Menten graph of Ldt_Mt2_ in reaction with substrate **1**. **D** Michaelis-Menten graph of Ldt_Mt2_ in reaction with substrate **3**.

Thus far, Ldt_Mt2_ transpeptidase activity has been measured by LC-MS analysis^13^, and Ldt_Mt2_ inhibition assays have relied on use of unnatural chromogenic or fluorogenic probe substrates^23,33,34^, as employed in an assay suitable for high-throughput screening^26^. These assays are, however, likely poor mimics of the natural reaction. We therefore developed an efficient, quantitative, and biologically relevant method of determining Ldt_Mt2_ activity and inhibition based on turnover of its natural substrate. A microtiter plate-based assay that determines PBP activity has been reported, based on detection of d-Ala, which is released on d,d-transpeptidase and d,d-carboxypeptidase activity (Fig. 3A)^35–37^. d-Ala is converted to pyruvate, ammonia and hydrogen peroxide by d-amino acid oxidase (DAAO). Hydrogen peroxide is then used by horseradish peroxidase (HRP) to oxidise Amplex Red (AR) forming fluorescent resorufin^35^. We considered that the transpeptidase assay could be extended to monitor Ldt_Mt2_ catalysis.

Optimisation initially involved variation of DAAO and HRP concentrations, aiming to minimise delay in conversion of d-Ala. When the assay mixture contained an excess of DAAO (2 U/mL) and HRP (2 U/mL), conversion of d-Ala was apparently near instantaneous (Fig. S12A, B). The assay performed better in sodium phosphate compared to Tris or HEPES buffers (Fig. S12C), and in the presence of 100 mM NaCl (Fig. S12D). A higher pH manifested increased Ldt_Mt2_ activity (tested pH range 7.0-8.5; Fig. S12E) and activity was higher at 37 °C than room temperature (Fig. S12F). Assays were therefore performed at 37 °C in 50 mM sodium phosphate pH 8.0, 100 mM NaCl and 0.01% (*v*/*v*) Triton X-100, with an Ldt_Mt2_ concentration of 500 nM and substrate (**3**) concentration of 35 µM (Fig. S12G). Under these conditions, a *Z*′ value of 0.88 and a signal to background value of 5.0 was obtained. The hydrogen peroxide produced has potential to oxidise the Ldt_Mt2_ catalytic cysteine, however, dose-response studies^34^ manifested no evidence for interference with assay relevant concentrations (Fig. S12H).

The assay was applied to investigate the inhibition of reported Ldt_Mt2_ inhibitors (Fig. S13 and Table S2)^26,27,33^. The results showed good correlation with the reported Ldt_Mt2_ ligand binding assay utilising a fluorogenic thiol reactive probe (Fig. 3B)^34^. The DAAO/HRP method presents an improved assay for Ldt_Mt2_ inhibitor screening, as the assay more closely resembles the biological process. The possibility for interference through inhibition of DAAO and HRP necessitates use of appropriate controls; indeed, the nonspecific inhibitors ebselen **24** and ebsulfur analogue **23** were found to cause interference (Fig. S14 and Table S2). Note that the assay could be adapted to use l-lactate dehydrogenase to monitor reduction of NAD^+^ in the presence of l-lactate.

With fixed concentrations of **1** and **3** (35 µM), a substantial difference in reaction rates was observed (120.0 ± 1.7 RFU min^−1^ and 224.2 ± 2.6 RFU min^−1^ for **1** and **3**, respectively; Fig. S15), suggesting that interaction of the GlcNAc-MurNAc moiety of the substrate, at least with isolated components, has the potential to hinder the reaction rate. Increasing concentrations of **1** did not saturate the reaction (up to 20 mM; Fig. 3C and Table S3), hence the K_m_ of **1** could not be determined. With **3**, a K_m_ of 6.2 mM was obtained, indicating low affinity; *k*_cat_ was 2.6 × 10^2 ^min^−1^, providing a *k*_cat_/K_m_ of 4.2 min^−1^ M^−1^ (Fig. 3D and Table S3). Note, that the effective incorporation of fluorescent amino acids and peptidoglycan fragments into the *Mtb* cell wall suggests more efficient turnover occurs in a cellular context^38^. The difference between isolated activity and cellular activity may reflect a need for complex formation, missing cofactors, or for binding of full-length peptidoglycan for optimal Ldt_Mt2_ activity.

The non-substrate peptidoglycan fragments **4,** **8,** **10,** **12** and **13** showed modest Ldt_Mt2_ inhibition (Fig. S16). Pentapeptides **8** and **10** and tripeptide **12** were the most potent (pIC_50_ 4.8), while dipeptide **13** manifested the lowest potency (pIC_50_ 4.0). The ability of structurally related non-substrate peptides to displace the substrate from the active site shows potential for the development of substrate-like inhibitors, although optimisation to improve affinity will be required.

### X-ray crystallographic studies of the Ldt_Mt2_-substrate complex

To investigate the structural interactions of Ldt_Mt2_ with its substrate, we carried out X-ray crystallographic studies with the peptidoglycan fragments, aiming to obtain a structure of an enzyme-substrate complex; we obtained structures of Ldt_Mt2_ in complex with **1** and **3**.

**Fig. 4:**
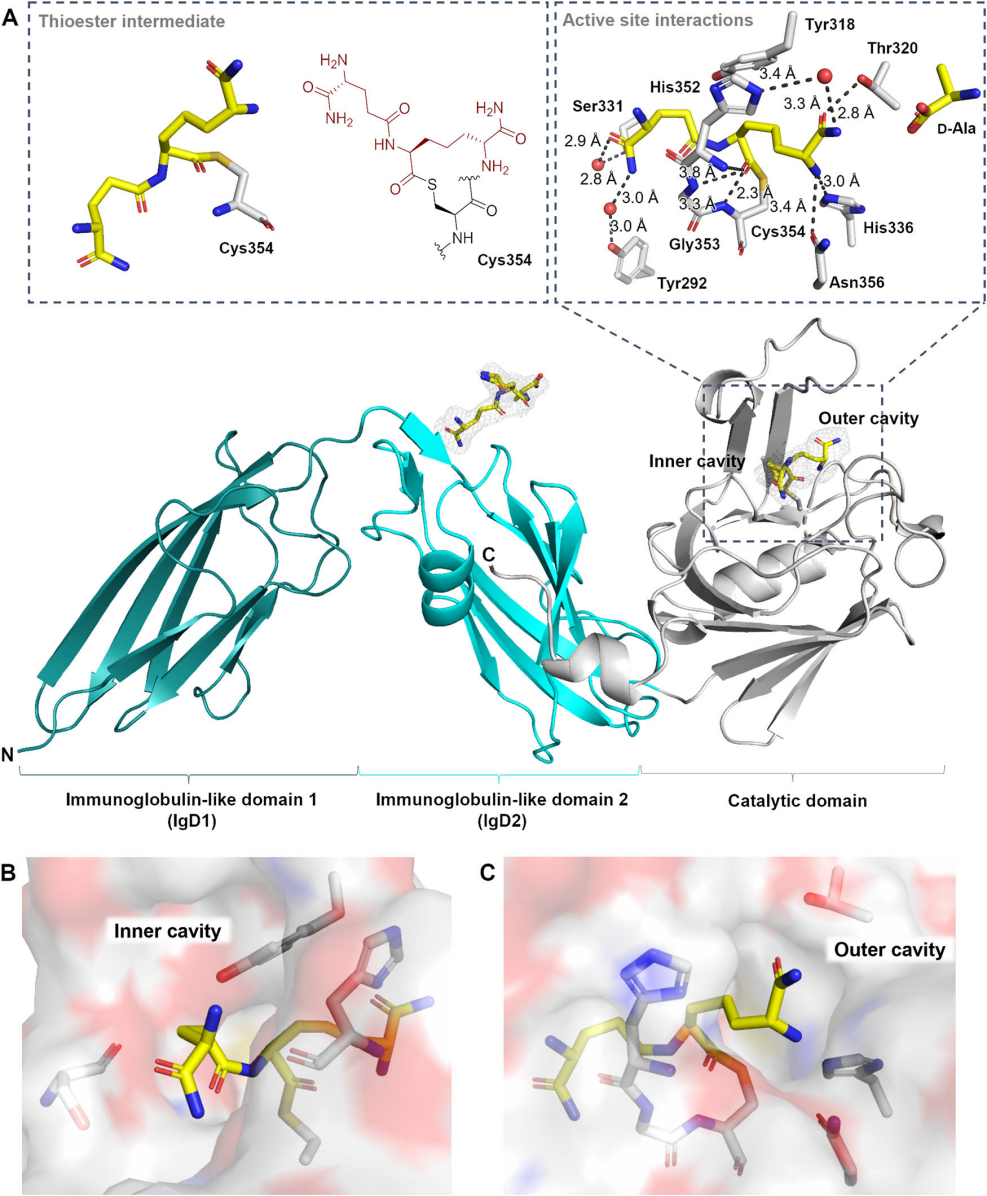
Crystallographic studies of Ldt_Mt2_ with peptidoglycan fragment 3 show covalent thioester formation. **A** A structure of Ldt_Mt2_ reacted with **3** (PDB 8PXZ, 1.98 Å resolution) shows the covalent thioester intermediate structure, and a second molecule of **3** bound to the IgD2 of Ldt_Mt2_. The *mF*_0_ − *DF*_c_ polder OMIT map is contoured at 3.0 σ and is carved around the **3**-derived thioester; **3** is in grey mesh. Polar interactions are shown in grey dashes. **B** View of the inner cavity, which interacts with the d-iGlu_NH2_ residue. **C** View of the outer cavity, which interacts with the *m*-DAP_NH2_ residue.

While in the absence of **3**, Ldt_Mt2_ crystallised in the reported *P*12_1_1 space group^26,27^, in the presence of **3** Ldt_Mt2_ crystallised in the *P*2_1_2_1_2 space group with a single protein chain in the asymmetric unit (1.98 Å, Fig. 4; PDB 8PXZ), using precipitation solutions with a pH between 7.0–7.5, yielding structures of Ldt_Mt2_ with a molecule of **3** bound to the Ldt_Mt2_ immunoglobulin-like domain 2 (IgD2). Notably, however, when Ldt_Mt2_ and **3** were co-crystallised using a precipitation solution at pH 7.0, we observed additional electron density extending from the sidechain of the nucleophilic residue Cys354. The results are in agreement with formation of the dipeptide (d-iGlu_NH2_-*m*-DAP_NH2_), covalently linked to Ldt_Mt2_ via a thioester with Cys354, apparently reflecting a key intermediate of the transpeptidase reaction with the donor peptide (Fig. 4A). The thioester is observed in the (*Z*)-stereochemistry, consistent with prior studies on nucleophilic serine- and cysteine-enzymes, including the SARS-CoV-2 main protease (M^pro^; Fig. S17A)^39,40^ and porcine pancreatic elastase^41^.

In the Ldt_Mt2_-**3** complex structure, the *m*-DAP_NH2_ residue sidechain protrudes into the outer cavity, while the d-iGlu_NH2_ sidechain is located in the inner cavity, suggesting that the donor substrate enters from the inner cavity (Fig. 4B, C). Note that no electron density was observed corresponding to the l-Ala residue of **3**, which has previously been suggested not to play a significant role in substrate binding^42^. Additional electron density at the entrance of the outer cavity was observed, in which d-Ala, released during the formation of the thioester intermediate, could be modelled (Fig. 4A). We did not observe density that could reflect binding of an acceptor substrate.

The overall fold of the Ldt_Mt2_-**3** complex strongly resembles that observed for unmodified Ldt_Mt2_ (PDB 6RLG^27^; main chain RMSD 0.65 Å). Similarly to unmodified Ldt_Mt2_^27,43^, the S^γ^ atom of Cys354 is positioned 3.9 Å from the N^ε2^ atom of His336, and the N^δ1^ atom of His336 is positioned for polar interactions with the main-chain carbonyl O of Ser337 at a distance of 2.7 Å. Notably, no conformational changes relative to unmodified Ldt_Mt2_ are observed in loop II (Fig. S18), which encloses the active site (residues 300-323) and which has previously been observed to undergo conformational changes upon binding of various Ldt_Mt2_ inhibitors, and which has been proposed to undergo extensive conformational changes upon substrate binding^26,33,43^.

The thioester acyl group, as well as the *m*-DAP_NH2_ residue and the d-iGlu_NH2_ residue of **3** are positioned to engage in interactions with elements in the Ldt_Mt2_-**3** complex active site (Fig. 4, S19). The thioester acyl group is located in the proposed oxyanion hole (the backbone NH groups of His352, Gly353 and Cys354) at respective distances of 3.8 Å, 3.3 Å and 2.3 Å, suggesting that, at least in the crystal structure obtained, no strong interactions are made with the backbone NH groups of His352. Polar interactions of *m*-DAP_NH2_ with His336 (3.0 Å), Asn356 (3.4 Å) and Thr320 (2.8 Å), as well as water-mediated polar interactions with His352 (3.3 Å to water, 3.4 Å to His352) are observed. The *m*-DAP_NH2_ carbon chain is positioned for hydrophobic interactions with the sidechain of Tyr318. The d-iGlu_NH2_ residue is observed to engage in water-mediated polar interactions with His352 (2.7 Å to water and 3.3 Å to His352) and Ser331 (2.9 Å to water and 2.8 Å to Ser331), as well as hydrophobic interactions with Gly332.

Crystallographic studies of Ldt_Mt2_ in the presence of **1** did not manifest evidence for the presence of substrate at the active site; similarly to **3**, however, **1** was observed to bind to the surface of the IgD2 domain through various polar interactions, as observed with both wild-type Ldt_Mt2_ and with Ldt_Mt2_^C354S^ (PDB 8PXY, 2.15 Å; Fig. S20). The GlcNAc moiety of **1**, which is absent in **3**, was observed to interact with the surface the catalytic domain of a second symmetry related molecule of Ldt_Mt2_, manifesting polar interactions with Asp367 (2.5 Å and 2.8 Å) and with Gln363 (3.0 Å), located ~20 Å away from the nucleophilic cysteine (or serine, in the case of Ldt_Mt2_^C354S^), potentially preventing binding of the second molecule of **1** at the Ldt_Mt2_ active side as observed in the structure of Ldt_Mt2_ co-crystallised with **3**.

## Discussion

Targeting bacterial cell wall transpeptidases is a validated method for treating bacterial infections, in particular via β-lactam mediated PBP inhibition^5^. Though a role for β-lactams in the widespread treatment of TB has not been established, promising activity against *Mtb* has been observed, particularly with carbapenems, which inhibit both PBPs and Ldts^44^. Ldts, and specifically Ldt_Mt2_, are essential for *Mtb*^13^, but have been considerably less intensively studied than PBPs. Whilst several early-stage studies on inhibitors for Ldt_Mt2_ are reported^23,26^, the search for potent and clinically useful inhibitors is ongoing. Despite its biological and medicinal importance, the mechanism of the transpeptidase reaction of Ldt_Mt2_ has been the subject of limited studies, in part likely due to the lack of availability of the peptidoglycan monomers. Reported studies on Ldt_Mt2_ have thus mainly relied on the use of relatively simple substrate analogues^22,45^. Our modified procedure for the isolation of peptidoglycan monomers from the *C. jeikeium* cell wall enabled us to obtain a crystal structure of Ldt_Mt2_ reacted with **3** and to obtain insights into the binding and reaction kinetics of Ldt_Mt2_.

Exposure of *C. jeikeium* cells to subinhibitory concentrations of ampicillin during growth, results in accumulation of peptidoglycan pentapeptide monomer **4**. Ldt_Mt2_ substrate **1** can subsequently be obtained from **4** via enzymatic cleavage of the terminal d-Ala. Via this method, the tetrapeptide monomer was obtained in a yield of ∼21 mg from 56 g of dried cell mass. Presently, this approach is substantially more efficient than reported synthetic procedures^28^.

X-ray crystallographic studies of Ldt_Mt2_ with substrate **3** yielded a structure of the covalently linked thioester acyl-enzyme complex, likely substantially reflecting the transient intermediate during the Ldt_Mt2_ transpeptidation reaction. Many enzymes employing nucleophilic cysteine- or serine-residues proceed through covalent acyl-enzyme intermediates; commonly these intermediates are unstable, and hence structures of them in complex with substrates, including at the acyl-enzyme complex stage, can be challenging to obtain under catalytically relevant conditions^46^. Crystallographic studies of the (thio)ester intermediates frequently rely on active site mutations (e.g., ref. ^47^), the use of substrate mimics (e.g., ref. ^48^), the use of rate-reducing conditions, for example, non-optimal pH (e.g., refs. ^41,49^), or use of an excess of a reversibly reacting product^41^. There is a particular lack of reported thioester, compared to ester, intermediate structures, perhaps reflecting the increased reactivity of thioesters with nucleophiles. To our knowledge, the only other nucleophilic cysteine enzyme where structural analysis of a thioester derived from a natural substrate has been achieved is SARS-CoV-2 M^pro^^39,40^, where a thioester was observed to form in crystallo after being presented with and excess of an acid product, resulting in reverse reaction.

The observation of a thioester in our Ldt_Mt2_-**3** complex acyl-enzyme crystal structure could in part be due to the inability of the acceptor substrate to enter the active site in the crystalline state due to reduced flexibility that may be required for acceptor substrate binding. In addition to the use of an excess of substrate (and hence product) and lattice interactions, other factors that may affect the persistence of the thioester in crystallo are the reduced pH (7.0, compared to 7.5) and temperature (4 °C, compared to rt) with respect to the solution-based studies, both of which reduce the rate of the Ldt_Mt2_ reaction (Fig. S12D-E). It is also important to note that transpeptidation reactions are fundamentally different from hydrolysis reactions, in that during transpeptidation the acyl-enzyme complex is required to be sufficiently stable in order to allow for the *m*-DAP residue of the acceptor substrate to react with the (thio)ester of the donating substrate.

The relative positions of the catalytic residues and of the oxyanion hole residues in the Ldt_Mt2_-**3** complex are similar to those observed in the M^pro^ thioester intermediate structure, and in structures of Ldts in complex β-lactams, e.g., in a structure of the *Enterococcus faecium* Ldt_fm_ complexed with ertapenem^50^, and in structures of Ldt_Mt2_ complexed with meropenem or biapenem (Fig. S17A–D)^21,51^. In all cases the thioester link has the (*Z*)-stereochemistry that is commonly associated with acyl-enzyme intermediates^46,52^, including in a structure of PBP4a of *B. subtilis* in complex with a peptidoglycan mimetic (Fig. S17E)^53^.

The structure reported here informs on how Ldt_Mt2_ interacts with a substantial part of its donor substrate. In a structure of a non-substrate peptidoglycan fragment (**13,** d-γ-Glu-*m*-DAP) bound to the Ldt_Mt2_ active site^22^, the *m*-DAP residue faces into the inner cavity, contrasting with our structure, suggesting entrance from the outer cavity (Figs. S21A, B and S22A). Molecular dynamics studies have suggested that entrance of the substrate from the inner cavity of Ldt_Mt2_ will not lead to acylation^42^. Our results suggest that during the initial steps of productive binding via the inner cavity, the substrate may project deeper into the active site than predicted by the reported calculations. In agreement with this proposal, a solid-state NMR structure of the *B. subtilis* l,d-transpeptidase (Ldt_Bs_) in complex with intact peptidoglycan suggests binding of the donor substrate in the analogous inner cavity (Figs. S21C, S22B)^54^.

An allosteric binding pocket of Ldt_Mt2_, named the S-pocket, is proposed to bind the sugar-moieties (Fig. S21E)^45^. We did not observe interaction of GlcNAc-MurNAc of **1** in this pocket. Nevertheless, extrapolation of the studies on Ldt_Bs_ bound to intact peptidoglycan suggests that intact peptidoglycan likely binds in the S-pocket associated with the IgD2 domain of Ldt_Mt2_^54^.

A coupled assay for detecting d-Ala was optimised for assessing Ldt_Mt2_ activity. The assay represents an increased throughput method of assessing turnover in comparison with previous LC-MS studies^13,28^, and provides increased biologically relevance over the reported high-throughput assay for inhibition of Ldt_Mt2_^26^. However, due to the relatively low enzymatic activity, high concentrations of substrate are required, making the use of this assay in a high-throughput setting challenging without a well-established synthesis route. Additionally, the coupled enzymes DAAO and HRP increase the risk of assay interference. Based on these observations, we recommend that the fluorescence-based transpeptidase assay is perhaps most suited for use as a secondary assay following more high-throughput assays for Ldt_Mt2_, testing potential inhibitors in a more biologically relevant manner.

Although our studies suggest that the isolated recombinant Ldt_Mt2_ has a relatively low affinity and catalytic activity with **1** and **3**, under our conditions **3** is a somewhat more efficient substrate. Interestingly, evidence for efficient transpeptidase activity by Ldts has been observed in *Mtb* cells in a previous report^38^, suggesting the need for increased interactions than present in our assays; potentially these will be achieved with use of full-length peptidoglycan. Alternatively, complex formation with related proteins / enzymes may increase activity, as observed with the functionally related d,d-transpeptidases and glycosyltransferases PBP1A and PBP2B, which require interactions with lipoprotein cofactors LpoA and LpoB, both in isolated enzyme and cellular systems^55–58^. In *E. coli*, the Ldt YcbB was found to associate with PBP1b and PBP5 revealing links between the two types of peptidoglycan transpeptidases^59^.

Amidation of both d-iGlu and *m-*DAP residues is reported to be required for Ldt_Mt2_ catalysis, as well as for growth of *Mtb*^28^. We observed that **11**, a close analogue of **3**, containing d-iGlu_NH2_ but with the *m-*DAP_NH2_ substituted with l-Lys, is not a substrate for Ldt_Mt2_. The Ldt_Mt2_-**3** complex structure suggests that the multiple polar interactions *m-*DAP_NH2_ makes with Asn356, His336, Thr320, and His352 are important, as l-Lys is likely only able to (directly) interact with Asn356 and His336. By contrast with the high substrate selectivity observed for Ldt_Mt2_, PBPs have been observed to react with d-Ala-d-Ala peptidoglycan mimetics, suggesting that modified features of the second and third peptide chain residues may not be as important for PBP catalysis as they appear to be for Ldt_Mt2_^60,61^.

Most reported Ldt_Mt2_ inhibitors interact with the catalytic cysteine (Cys354), and may fill either the inner or the outer active site cavities, e.g., in the Ldt_Mt2_:ertapenem complex the inhibitor occupies the inner cavity, while in the Ldt_Mt2_:biapenem complex the inhibitor occupies the outer cavity (Fig. S17C-D)^21,43,45,51^. Based on the structure of Ldt_Mt2_ reacted with **3**, elements of which occupy both the inner- and outer-cavities, improvement of inhibitor potency might be achieved through interactions with both the inner- and outer-cavities. Design of inhibitors achieving this may be based on our tetrapeptide structure, but optimisation will be necessary, especially in light of the low affinity of Ldt_Mt2_ for **3** in solution. The structure of Ldt_Mt2_ reacted with **3** suggests that a focus for future optimisation studies may be on the d-iGlu_NH2_-*m-*DAP_NH2_-d-Ala residues, in particular the d-iGlu_NH2_-*m-*DAP_NH2_ elements which are observed to interact with Ldt_Mt2_ in the intermediate structure. The addition of an electrophilic group to the *m-*DAP_NH2_ residue *in lieu* of the peptide bond with d-Ala may increase reactivity, e.g., a nitrile group, which has been shown to react with Cys354 of Ldt_Mt2_^26^. Optimisation of the substrate for inhibition, including the addition of a reversibly covalently reacting nitrile group has been a successful method in the inhibitor development of M^pro^, with one resulting inhibitor, nirmatrelvir, being approved for COVID-19 treatment^62,63^. Polar interactions with Tyr308 may be achieved through incorporation of a heteroatom in the d-iGlu_NH2_ carbon chain. While the carbon chain of the *m*-DAP_NH2_ does not undergo direct interactions with the active site of Ldt_Mt2_, the flexibility of this residue may be required to allow for entrance into the active site and the polar interactions observed with His336, Tyr320, and Asn356, as well as the water-mediated interactions with His352.

## Conclusion

Ldt_Mt2_ is a promising drug target for TB treatment, though little has been reported on the mechanism of the transpeptidase reaction that it catalyses. We optimised the isolation of the natural disaccharide tetrapeptide monomers **1**-**4** from the *C. jeikeium* bacterial cell wall through overproduction of peptidoglycan sacculus. We explored the interactions of Ldt_Mt2_ with the isolated tetrapeptide monomers **1** and **3** via X-ray crystallographic studies, obtaining a structure of the proposed thioester intermediate in Ldt_Mt2_ catalysis. The structure informs on the mode of entrance of the donor substrate into the Ldt_Mt2_ active site and active site interactions made by it. The combined results will be useful in the design of novel Ldt_Mt2_ inhibitors, including those based on substrate binding interactions, a strategy that has been successfully employed for other nucleophilic cysteine enzymes, including M^pro^^62,63^.

## Methods

### Materials

Faropenem was from Fluorochem Ltd., FAD was from Alfa Aesar, AR was from Cambridge Bioscience Ltd., Fmoc- d-iGln-OH was from Ambeed, **12** and **13** were from InvivoGen. **16**-**23** were obtained from the GSK HTS compound library. All other compounds were from Merck.

### Protein production and purification

Recombinant Ldt_Mt2_ was produced in *Escherichia coli* BL21(DE3) cells transformed with pNIC28-Bsa4-Ldt_Mt2_ Δ1-55, in the presence of 50 μg/mL kanamycin^31^. Expression was induced with isopropyl β-d-thiogalactopyranoside (IPTG, 0.5 mM) at OD_600_ of 0.6, with subsequent incubation overnight at 18 °C. Cells were lysed by sonication (SONIC Vibra-Cell, 60% amplitude) in the presence of DNase I; lysates were loaded onto a 5 mL HisTrap column (GE Life Sciences), pre-equilibrated in HisTrap buffer A (50 mM Tris, pH 8, 500 mM NaCl, 20 mM imidazole). The protein was eluted with HisTrap buffer B (50 mM this, pH 8, 500 mM NaCl, 500 mM imidazole) using a gradient from 0% to 100% buffer B. Protein-containing fractions were combined and the buffer was exchanged for HisTrap buffer A. The HisTag was cleaved using the TEV protease at 4 °C, over 12 h. The HisTag cleaved Ldt_Mt2_ was passed through a 5 mL HisTrap Column, and loaded onto a 300 mL Superdex 75 column (GE Life Sciences) pre-equilibrated in buffer C (50 mM Tris, pH 8, 500 mM NaCl). Ldt_Mt2_ was eluted with buffer C. The purity and identity of Ldt_Mt2_ was confirmed by SDS-PAGE (>95% purity) and MS (calculated 37975 Da, observed 37978 Da) analyses. Site-directed mutagenesis was carried out using the partial overlapping primer design method^64^, with primer sequences shown in Table 1 being used to achieve the C354S mutation of Ldt_Mt2_. PCR was performed using Q5 High-Fidelity DNA Polymerase (New England Biolabs) following standard protocols. Ldt_Mt2_^C354S^ was produced and purified as described for the wild-type enzyme^31^. Purity and identity of Ldt_Mt2_^C354S^ was confirmed by SDS-PAGE (>95% purity) and MS (calculated 37959 Da, observed 37962 Da). Recombinant *E. coli* DacA was produced and purified as reported^65^.

**Table 1:** Primers used in the site-directed mutagenesis to achieve the C354S mutation of Ldt_Mt2_

Primer	Sequence
Ldt_Mt2_^C354S^-forward	CAGCCATGGT**A**GTCTGAATGTTAGCCCGAGCAATG
Ldt_Mt2_^C354S^-reverse	CTAACATTCAGAC**T**ACCATGGCTGGTATTGGTATG

In bold is the nucleic acid that is changed from the wild type.

### Peptidoglycan fragment isolation

*C. jeikeium* (National Collection of Type Cultures: NCTC 11913) was grown overnight at 37 °C on Columbia agar supplemented with 5% sheep blood (Scientific Laboratory Supplies Ltd). Cultures were used to inoculate brain heart infusion broth supplemented with 1% (*v*/*v*) Tween 80 and 2 μg/mL ampicillin^29^. The culture was incubated at 37 °C for 16 hours with shaking.

Cells were collected by centrifugation (15,000×*g*, 20 min, 4 °C). The cell pellet was resuspended (1 mL/g) in 10 mM sodium phosphate pH 7.0 and lysed using a high-pressure cell disruptor (CF1, Constant Systems, 40 kpsi, 3x). The cell wall was collected by centrifugation (15,000×*g*, 15 min, 4 °C) and resuspended in 25 mL of 10 mM sodium phosphate pH 7.0. The resulting suspension was added dropwise to 25 mL of 10 mM sodium phosphate pH 7.0 with 8% (*w*/*v*) SDS and incubated for 30 min at 100 °C. Peptidoglycan was pelleted by centrifugation (45,000×*g*, 30 min, 15 °C), washed with 10 mM sodium phosphate pH 7.0 (5x), and treated with Pronase E (200 µg/mL, overnight at 37 °C with shaking, in 10 mM Tris-HCl pH 7.4). Next, the peptidoglycan was pelleted by centrifugation (45,000×*g*, 30 min, 15 °C) and was washed with 20 mM sodium phosphate pH 7.8, before treatment with trypsin (200 µg/mL, overnight at 37 °C with shaking, in 20 mM sodium phosphate pH 7.8). Peptidoglycan was washed twice with water and dissolved in 25 mM sodium phosphate pH 6.0 with 0.1 mM MgCl_2_ and treated with mutanolysin (200 µg/mL) and lysozyme (200 µg/mL) and incubated overnight at 37 °C with shaking. Insoluble peptidoglycan was pelleted by centrifugation (4000×*g*, 30 min, 4 °C). The supernatant containing soluble disaccharide peptides was collected.

The disaccharide peptides were separated via reverse phase high-performance liquid chromatography (HPLC) on a C_18_ column (10 µm, 21,2 × 250 mm, Avantor ACE AQ) at a flowrate of 15 mL/min. A gradient of 1-20% (*v*/*v*) of solvent B was applied between 0 and 20 min (solvent A: 0.05% (*v*/*v*) trifluoroacetic acid in water; solvent B: 0.035% (*v*/*v*) trifluoroacetic acid in acetonitrile). Peaks containing **4** were lyophilised and dissolved in 50 mM tris pH 7.5 and DacA (20 µM) was added. The mixture was incubated for 5 h at 37 °C, lyophilised and purified by HPLC on a C_18_ column (10 µm, 21,2 × 250 mm, Avantor ACE AQ) at a flowrate of 15 mL/min. A gradient of 1-5% (*v*/*v*) of solvent B was applied between 0 and 7.5 min, followed by a gradient of 5–7% (*v*/*v*) of solvent B over 40 min to obtain **1** as a white solid.

To obtain **2**, fragment **1** was dissolved in 0.5 M borate buffer pH 8.0 containing 10 mg/mL sodium borohydride. After 20 min the pH was adjusted to 2-4 with orthophosphoric acid. Purification by HPLC was performed as described above.

To obtain **3,** fragment **1** was dissolved in 25% (*v*/*v*) NH_4_OH in H_2_O and stirred at 37 °C for 5 hours. The mixture was then neutralised via addition of acetic acid and the mixture was lyophilised. Purification by HPLC was performed as described above.

### Syntheses of 9 and 11

**9** and **11** were synthesised by solid phase peptide synthesis using a Liberty Blue Automated Microwave Peptide Synthesizer. d-Alanine-Wang resin (715 mg, 0.5 mmol) was coupled with Fmoc protected amino acids d-Ala (1.5 mmol, 3 eq.), l-Lys (2.5 mmol, 5 eq.), d-iGlu_NH2_ (2.5 mmol, 5 eq.), and l-Ala (2.5 mmol, 5 eq.), respectively, in the case of **9**, and l-Lys (2.5 mmol, 5 eq.), d-iGlu_NH2_ (2.5 mmol, 5 eq.), and l-Ala (2.5 mmol, 5 eq.), respectively in the case of **11**. Coupling using the standard procedure was performed with *N,N*-diisopropylethylamine (DIPEA) in ethyl cyanohydroxyiminoacetate (Oxyma, 1 M; DIPEA/Oxyma 1.5% (*v*/*v*)) and 7.8% (*v*/*v*) *N,N*′-diisopropylcarbodiimide (DIC) in DMF. After each coupling step, the resin was washed with DMF and the Fmoc group was deprotected with 20% (*v*/*v*) piperidine in DMF using the standard deprotection procedure. After coupling and deprotection steps were repeated as required, the resin-bound peptide was washed with dichloromethane (DCM), methanol and DCM, and treated with a 5 mL solution of 2:1 TFA/DCM (2 h, rt). Solvent was removed by evaporation *in vacuo* and purified by HPLC on a C_18_ column (5 µm, 10 × 150 mm; SunFire, Waters) at a flowrate of 3 mL/min in 98% (*v*/*v*) buffer A (0.1% (*v*/*v*) formic acid in H_2_O) and 2% buffer B (0.1% (*v*/*v*) formic acid in acetonitrile).

### Mass spectrometry assays

LC-MS assays were performed using a 1290 Infinity II LC System (Agilent Technologies) equipped with a C_18_ column (3 µm, 50 × 2.1 mm; Ace equivalence) with a gradient of 5-20% (*v*/*v*) water:acetonitrile 0.1% (*v*/*v*) formic acid over 0 and 20 min, coupled to an Agilent 6550 iFunnel Q-TOF mass spectrometer (Agilent Technologies) operating in positive mode. Data were analysed using MassHunter Qualitative Analysis B.07.00 (Agilent Technologies).

Protein-observed SPE-MS assays were performed with a RapidFire200 integrated autosampler/solid phase extraction (SPE) system (Agilent Technologies) coupled to an Agilent 6550 iFunnel Q-TOF mass spectrometer (Agilent Technologies) operating in the positive ionisation mode^26^. Ldt_Mt2_ (1 µM) in 50 mM tris pH 7.5 was incubated with peptidoglycan fragments (100 µM), loaded onto a C4 cartridge (Aligent Technologies), and eluted with organic phate (85% (*v/v*) acetonitrile, 15% (*v/v*) water, 0.1% (*v/v*) formic acid. Data were analysed using MassHunter Qualitative Analysis B.07.00 (Agilent Technologies).

### Transpeptidase assays

A transpeptidase assay master mixture was prepared in assay buffer (50 mM sodium phosphate, pH 8.0, 100 mM NaCl, 0.01% (*v/v*) Triton X-100) as described in Table 2. The specified master mixture (14 µL) was added to a black polystyrene, clear- and flat-bottomed, 384-well microplate (Greiner Bio-One, part number 781096) using a MultiDrop Combi dispenser (Thermo Fisher Scientific).

**Table 2:** Master mixture for the DAAO assay

Component	Concentration (1.8x)	Final concentration
DAAO	3.6 U/mL	2.0 U/mL
FAD	72 µM	40 µM
HRP	3.6 U/mL	2.0 U/mL
Amplex red	18 µM	10 µM

Compounds (in DMSO, with final concentrations ranging from 40 µM to 20.3 nM; 10 dilutions of factor 3) were added using a CyBio liquid handling system (Analytik Jena AG), with 4 replicates per inhibitor concentration. In the case of Control 1 (expected 0% inhibition; 100% fluorescence signal) and Control 2 (expected 100% inhibition; 0% fluorescence signal) the compound solution was substituted with neat DMSO. Ldt_Mt2_ (5 µL, 500 nM final concentration) was added to all wells using a MultiDrop Combi dispenser (Thermo Fisher Scientific). In the case of Control 2, Ldt_Mt2_ was substituted with the assay buffer (5 µL). This mixture was incubated for 30 minutes at rt. The substrate (**1** or **3**, as specified, 5 µL, 35 µM final concentration) was then added to all wells. The fluorescence signal was measured using a PHERAstar plate reader (BMG Labtech) with λ_ex_ = 540 nm and λ_em_ = 590 nm. Data were analysed using Prism 10 (GraphPad).

The transpeptidase interference assay was performed as described above, with the modification that the Ldt_Mt2_ mixture was replaced by 5 µL assay buffer, and the substrate was replaced by d-Ala (500 nM final concentration). Data were analysed using Prism 10 (GraphPad).

### Crystallography

Ldt_Mt2_ wild type or Ldt_Mt2_^C354S^ (Δ1-55; with the N-terminal His Tag removed, in 50 mM tris, pH 8.0, 100 mM NaCl) was incubated with substrate **1** or **3** for 30 minutes on ice, and then crystallised using a well solution consisting of 0.1 M HEPES, pH 7.0, and 25% (*v*/*v*) Jeffamine ED-2001 pH 7.0 (identified from the JCSG Plus^TM^ crystallisation screen, Hampton Research), using sitting drop vapour diffusion crystallisation plates (low reservoir Intelli-Plate 93-3), with 1 µL of protein-substrate solution and 1 µL of well solution. Note that crystallisation conditions previously reported by us^26,27^ did not yield crystals with additional electron density in the active site upon either soaking existing crystals with substrates **1**-**3** or cocrystallisation with **1**-**3**, neither did we obtain complexes with Ldt_Mt2_^C354S^. Crystallisation plates were stored at 4 °C, and crystals grew over 24 h. The crystals were mounted on nylon loops, cryocooled and stored in liquid nitrogen. Datasets were collected using the MX beamline i03 at Diamond Light Source at 100 K, at a wavelength of 0.97625 Å. Datasets were processed using the automated processing pipeline at Diamond Light Source, using xia2^66^. Structures were solved by molecular replacement with Phaser^67^, using PDB entry 6RLG^27^ as the search model. Ligand restrain files were created using Grade2^68^. Alternating cycles of refinement using PHENIX^69^ and manual model building using COOT^70^ were performed until no further significant improvement of *R*_work_ and *R*_free_ was achieved. Data collection and refinement statistics are described in Table 3. There were no Ramachandran outliers for the Ldt_Mt2_^C354S^-**1** (PDB 8PXY) and Ldt_Mt2_-**3** (PDB 8PXZ) structures, with 98% and 97% Ramachandran favoured, respectively.

**Table 3:** Data collection and refinement statistics for the crystal structure of Ldt_Mt2_^C354S^ in complex with 1 and Ldt_Mt2_ in complex with 3

	Ldt_Mt2_^C354S^ – 1 (PDB: 8PXY)	Ldt_Mt2_ – 3 (PDB: 8PXZ)
Data collection		
Space group	*P* 2_1_ 2_1_ 2	*P* 2_1_ 2_1_ 2
Cell dimensions		
*a*, *b*, *c* (Å)	76.61, 94.62, 58.79	77.12, 98.83, 59.23
*α*, *β*, *γ* (°)	90.00, 90.00, 90.00	90.00, 90.00, 90.00
Resolution (Å)	58.79–2.15 (2.23–2.15)	42.18–1.98 (2.05–1.98)
* R*_merge_	0.138 (0.987)	0.135 (1.997)
* I*/σ*I*	10.8 (1.1)	12.3 (1.4)
Completeness (%)	100.0 (97.0)	99.6 (94.2)
Redundancy	13.2 (13.5)	13.2 (11.1)
Refinement	PHENIX	PHENIX
Resolution (Å)	58.79–2.15 (2.23–2.15)^a^	42.18–1.98 (2.05–1.98)^a^
No. reflections	23,889 (2322)	31,078 (2883)
* R*_work_/*R*_free_	0.2032/0.2451	0.1848/0.2259
No. atoms	3073	3069
Protein	2655	2664
Ligand/ion	81	53
Water	337	352
* B*-factors	39.90	41.17
Protein	39.40	40.14
Ligand/ion	52.91	68.86
Water	40.73	44.86
R.m.s. deviations		
Bond lengths (Å)	0.003	0.010
Bond angles (°)	0.55	0.86

^a^A single crystal was used for each structure. Values in parentheses are for the highest-resolution shell.

### Statistics and reproducibility

All experiments were performed 2-4 times, as indicated. Individual data points are plotted, and average values and standard deviation are presented in corresponding tables.

### Reporting summary

Further information on research design is available in the Nature Portfolio Reporting Summary linked to this article.

## Supplementary information

Supplementary information

Description of Additional Supplementary Materials

Supplementary Data 1

Reporting summary

## Data Availability

The authors declare that the data that supports the findings of this study are available within the paper [and its supplementary files]. Source data for all figures and tables are available within the Supplementary Data file. Crystallographic data that supports the findings of this study have been deposited in the Protein Data Bank (PDB) with the accession codes “8PXY” and “8PXZ”. Requests for data should be sent to Christopher J. Schofield (christopher.schofield@chem.ox.ac.uk).
