# Supplementary material for: Biochemical and crystallographic studies of l,d-transpeptidase 2 from Mycobacterium tuberculosis with its natural monomer substrate
Source: Commun Biol. 2024 Sep 18;7:1173. doi: 10.1038/s42003-024-06785-3 (PMC11410929; doi:10.1038/s42003-024-06785-3)
Supplement: Supplementary file 1 — Supplementary information [file 42003_2024_6785_MOESM1_ESM.pdf]

Supplementary information

Biochemical and crystallographic studies of L,D-transpeptidase 2 from *Mycobacterium tuberculosis* with its natural monomer substrate

Mariska de Munnik,[1] Pauline A. Lang,[1] Karina Calvopiña,[1] Patrick Rabe,[1] Jürgen Brem,[1,2] and Christopher J. Schofield[1*]

[1] Chemistry Research Laboratory, Department of Chemistry and the Ineos Oxford Institute of Antimicrobial Research, University of Oxford, 12 Mansfield Road, Oxford, OX1 3TA, United Kingdom.

[2] Current address: Enzymology and Applied Biocatalysis Research Center, Faculty of Chemistry and Chemical Engineering, Babes-Bolyai University, Str. Arany Janos, nr. 11, Cluj-Napoca, RO-400028, Romania.

* Email: christopher.schofield@chem.ox.ac.uk

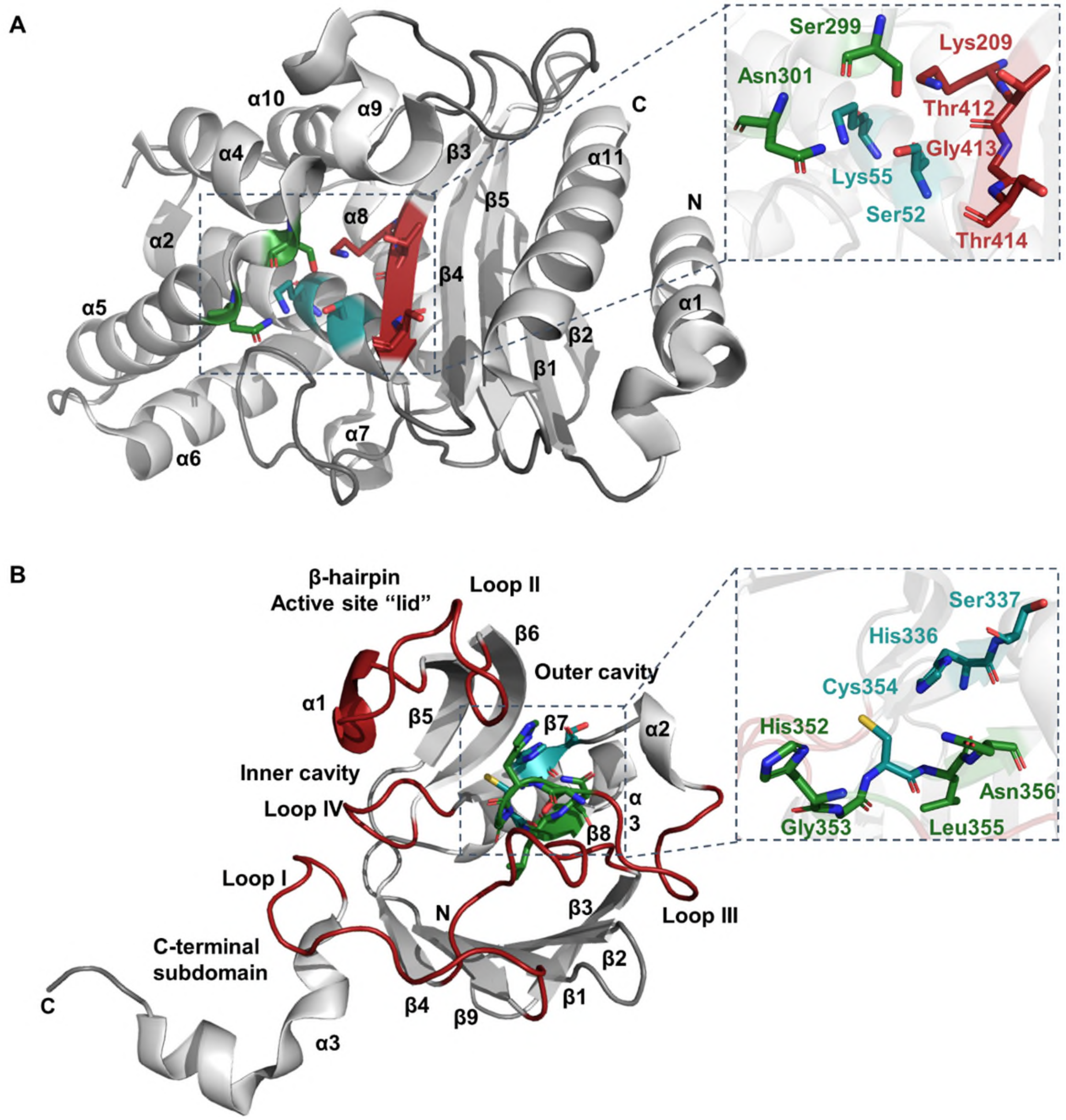

Figure S1. Views from structures of the transpeptidase domain of a representative D,D-transpeptidase (*Bacillus subtilis* PBP4a) and a representative L,D-transpeptidase (*Mycobacterium tuberculosis* Ldt$_{\mathrm{Mt2}}$). A. View of *B. subtilis* PBP4a (PDB: 1W5D),[1] showing the common peptidase domain fold of PBPs (grey), as described by Sauvage *et al.*[2] The close-up shows the highly conserved catalytic dyad SxxK motif (cyan) containing the nucleophilic serine, the SxN motif (green) that is involved in protonation of the β-lactam leaving group, and the KTG(S/T) motif (red) that is involved in substrate binding and forming an oxyanion hole. **B.** View of the *M. tuberculosis* Ldt$_{\mathrm{Mt2}}$ (PDB: 6RLG),[3] showing the common YkuD catalytic domain fold of Ldts (grey) and the conserved Hxx$_{14–17}$(S/T)HGChN motif (green). The catalytic triad residues (nucleophilic Cys354, His336 and Ser337) are in teal and loops I-IV (red), which are not highly conserved between Ldt variants.

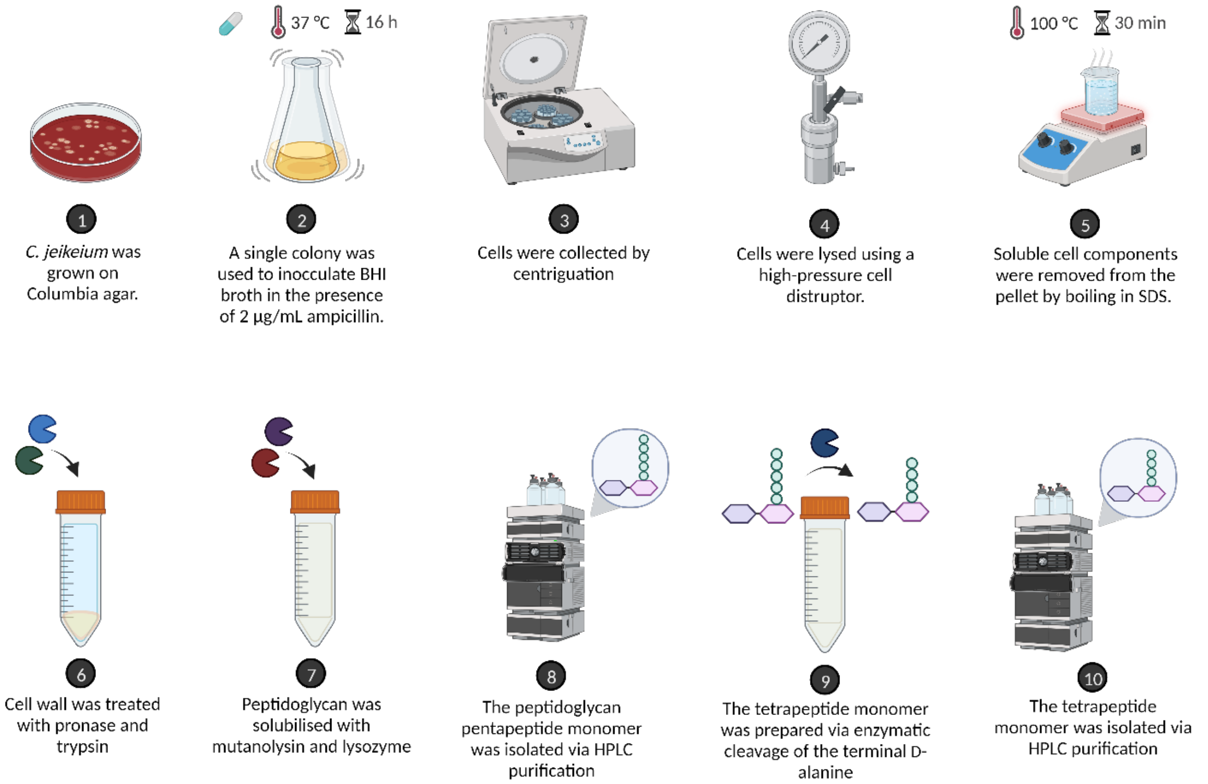

Figure S2. Method for the isolation of the tetrapeptide disaccharide monomer from the cell wall of *Corynebacterium jeikeium*. See Methods for experimental details. The figure was created using BioRender.com.

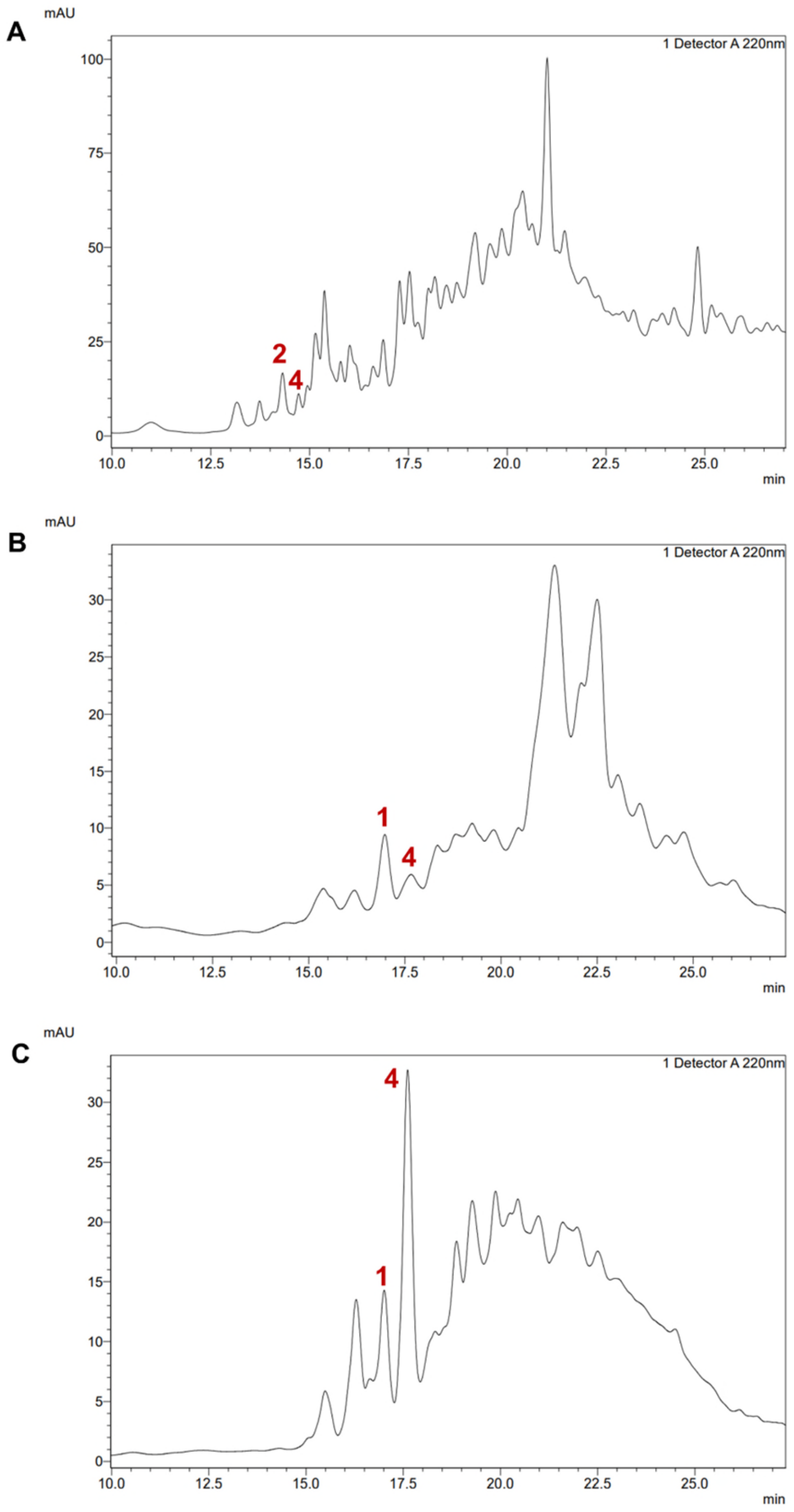

Figure S3. HPLC profiles of isolated peptidoglycan from lysed *Corynebacterium jeikeium* cells. A. HPLC profile of soluble peptidoglycan, where MurNAc has been reduced to muramitol via treatment of soluble peptidoglycan with sodium borohydride. **B**. HPLC profile of soluble peptidoglycan, omitting the MurNAc reduction step. **C**. HPLC profile of soluble peptidoglycan, when *C. jeikeium* was exposed to ampicillin, leading to overproduction of disaccharide pentapeptide. Structures of the numbered fragments are given in Figure 2. See Methods for experimental details.

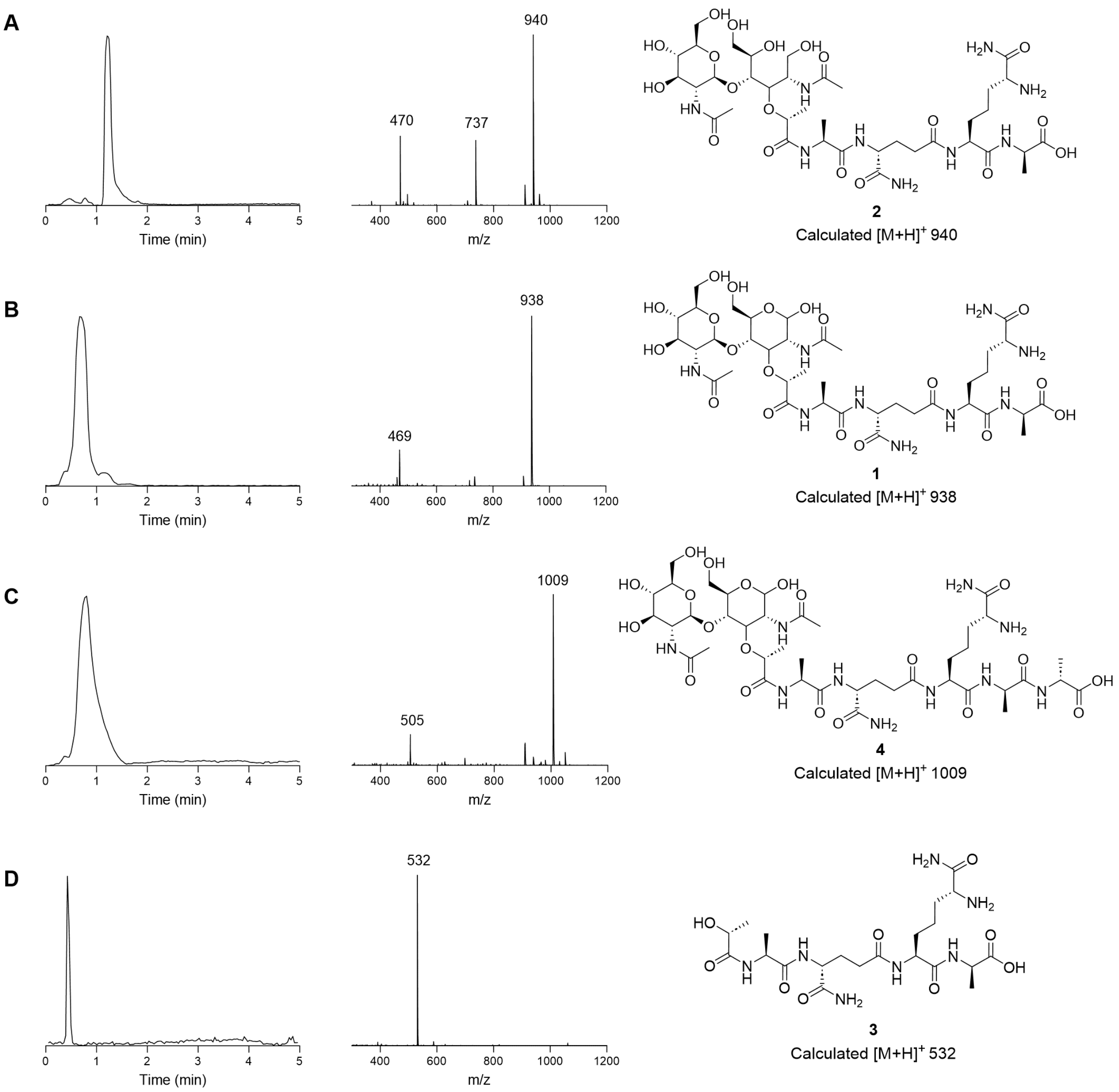

Figure S4. Liquid chromatography-mass spectrometry (LCMS) of peptidoglycan fragments isolated from lysed *Corynebacterium jeikeium* cells. A. LC (left) and MS (right) analyses of the isolated reduced disaccharide tetrapeptide **2**. **B**. LC (left) and MS (right) analyses of the isolated disaccharide tetrapeptide **1**. **C**. LC (left) and MS (right) analyses of the isolated disaccharide pentapeptide **4**. **D.** LC (left) and MS (right) analyses of the lactoyl tetrapeptide **3**, obtained from reaction with **1** (Figure S6). See Methods for experimental details.

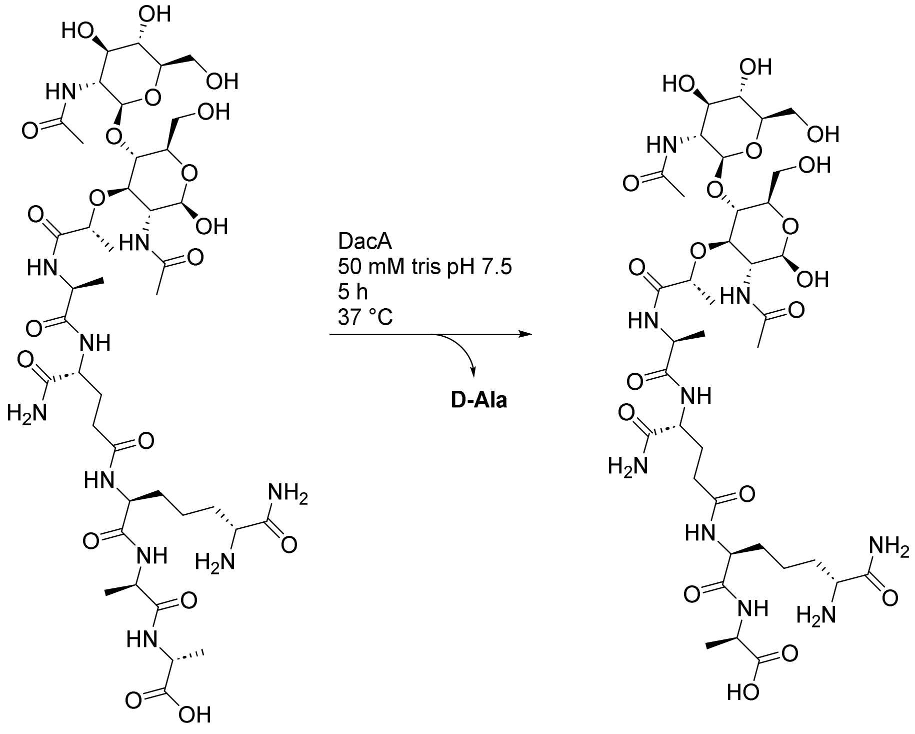

Disaccharide pentapeptide (4) **Disaccharide tetrapeptide (1)**

Figure S5. Reaction scheme for formation of disaccharide tetrapeptide 1 from disaccharide pentapeptide 4. The disaccharide pentapeptide **4** was dissolved in 50 mM tris buffer, pH 7.5. To this solution was added the *E. coli* D,D-carboxypeptidase DacA (20 µM). The mixture was incubated for 5 h at 37 °C. See Methods for experimental details.

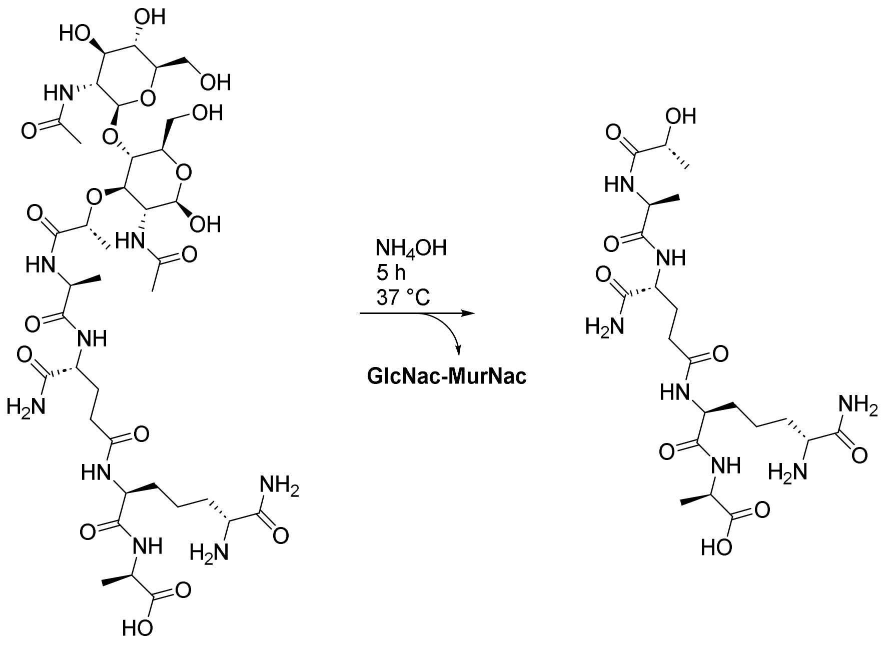

Disaccharide tetrapeptide (1)

Lactoyl tetrapeptide (3)

Figure S6. Reaction scheme for formation of lactoyl tetrapeptide 3 from disaccharide tetrapeptide 1. The disaccharide tetrapeptide **1** was dissolved in 25% (*v/v*) NH_4OH in H_2O and incubated for 5 h at 37 °C. See Methods for experimental details.

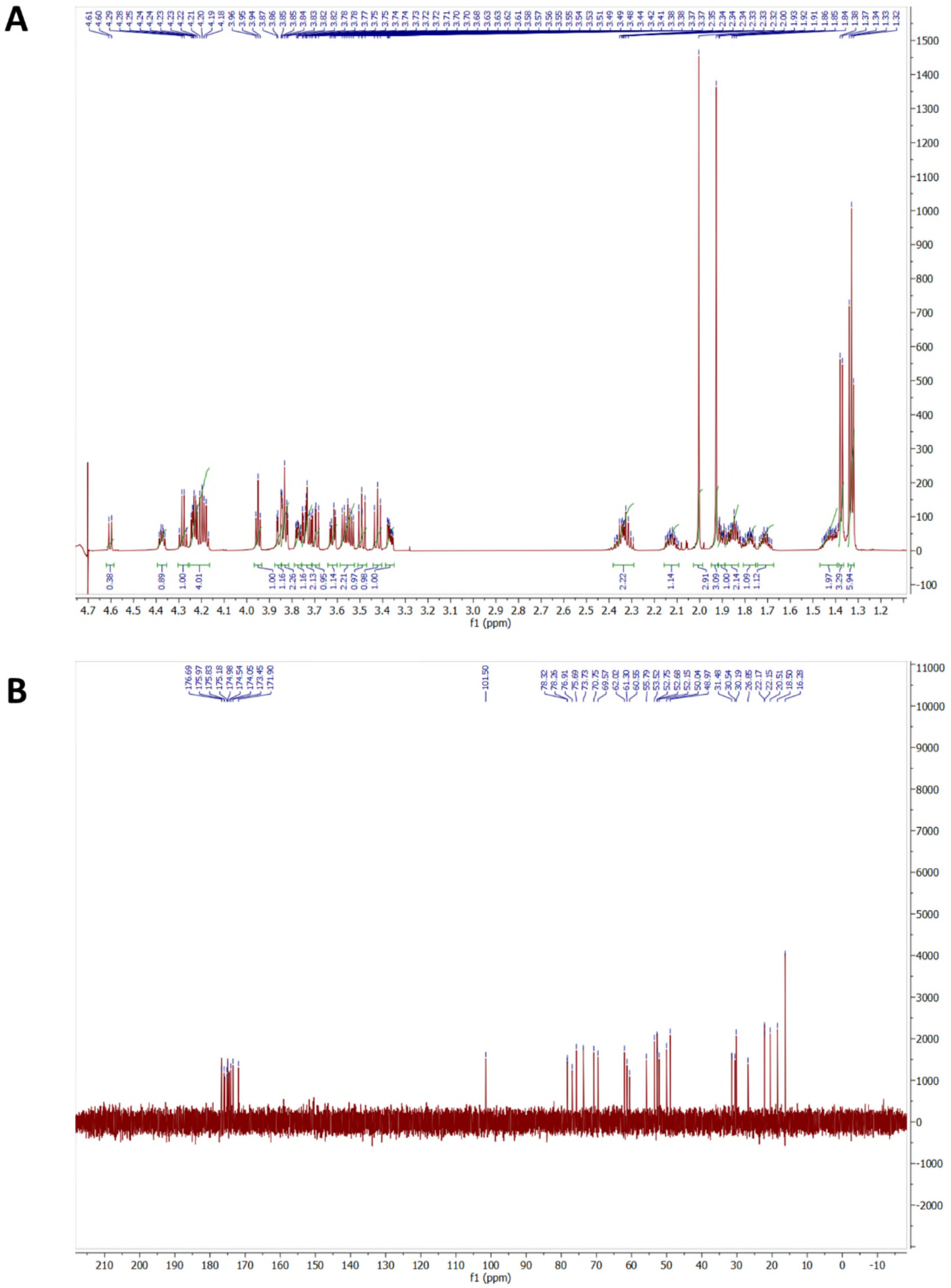

Figure S7. NMR characterisation (700 MHz) of substrate 2 in D_2O. [continues]

C

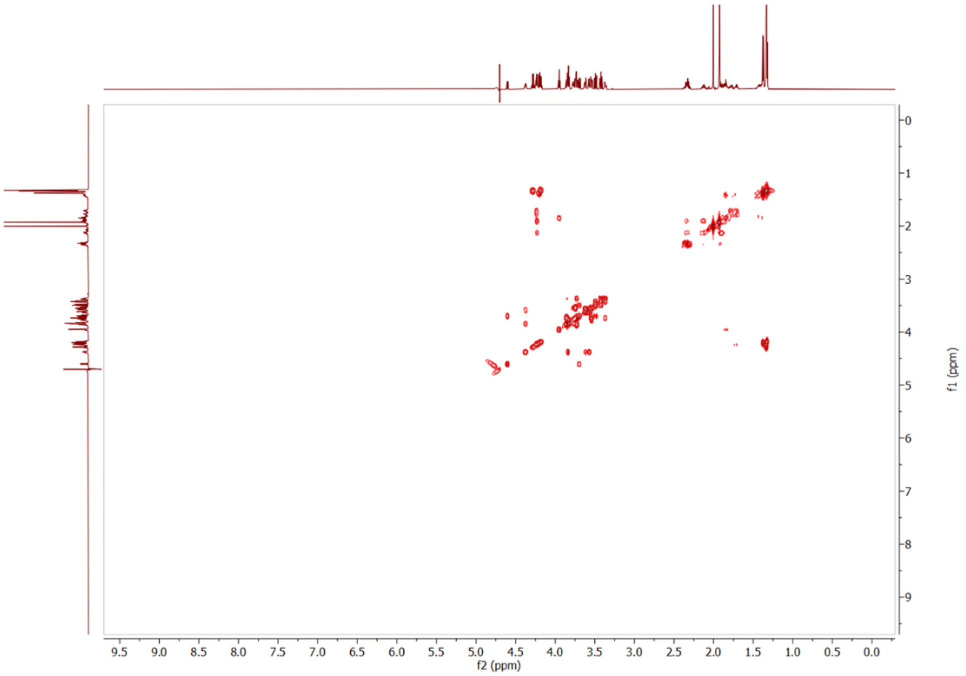

D

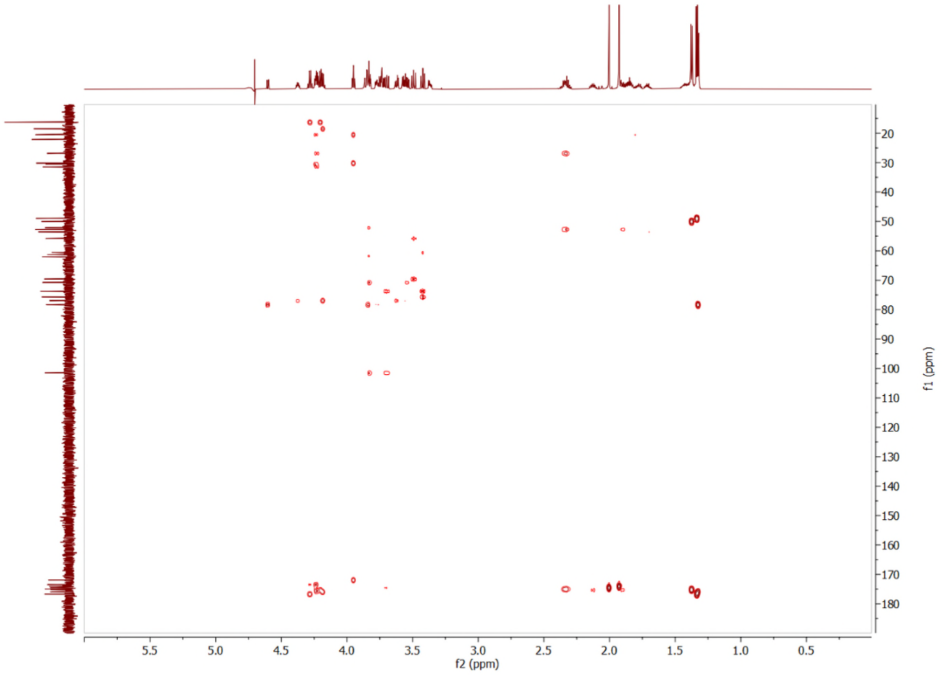

Figure S7. NMR characterisation (700 MHz) of substrate 2 in D_2O. [continues]

E

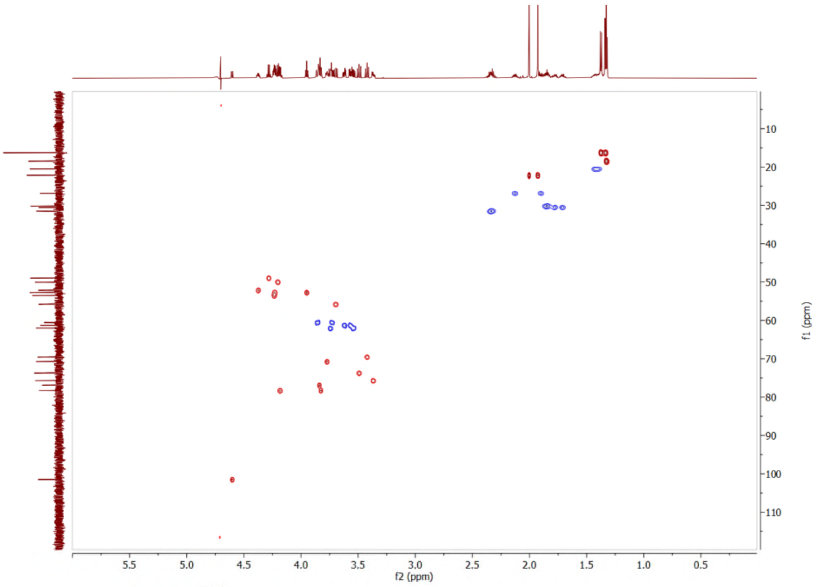

F

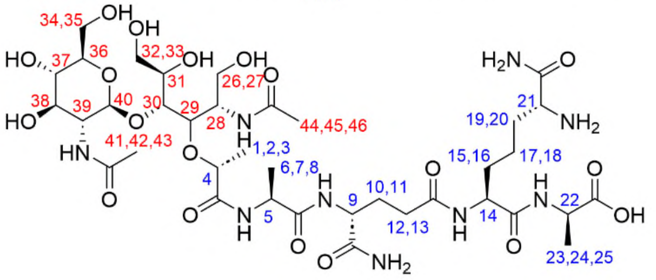

2

Chemical shift (ppm)	Multiplicity	Integration	Assignment
4.39 – 4.36	m	1H	28,29,30,31
4.28	q (*J* = 7.3 Hz)	1H	22
4.25 – 4.16	m	4H	4,5,9,14
3.95	t (*J* = 6.6 Hz)	1H	21
3.88 – 3.81	m	3H	28,29,30,31,34,35
3.79 – 3.76	m	1H	28,29,30,31
3.75 – 3.71	m	2H	26,27,32,33,34,35
3.71 – 3.68	m	1H	28,29,30,31
3.64 – 3.60	m	1H	26,27,32,33
3.59 – 3.53	m	2H	26,27,32,33,34,35
3.49	dd (*J* = 11.9, 4.4 Hz)	1H	36,37,38,39,40
3.44 – 3.40	m	1H	36,37,38,39,40
3.38 – 3.35	m	1H	36,37,38,39,40
2.38 – 2.29	m	2H	10,11
2.16 – 2.09	m	1H	12,13
2.00	s	3H	41,42,43,44,45,46
1.93	s	3H	41,42,43,44,45,46
1.92 – 1.89	m	1H	12,13
1.88 – 1.82	m	2H	19,20
1.81 – 1.75	m	1H	15,16
1.74 – 1.68	m	1H	15,16
1.47 – 1.39	m	2H	17,18
1.38	d (*J* = 7.3 Hz)	3H	6,7,8
1.35 – 1.32	m	6H	1,2,3,23,24,25

Figure S7. NMR characterisation (700 MHz) of substrate 2 in D_2O. NMR analyses of **2**, obtained from isolation of the cell wall of *C. jeikeium*, comprised of ^{1}H (**A**), ^{13}C (**B**), ^{1}H COSY (**C**), ^{1}H-^{13}C HMBC (**D**) and ^{1}H-^{13}C HSQC (**E**). **F**. Structure of **2** labelled with assignments. Note that assignment of the GlcNAc-muramitol unit (red assignments) is complicated by overlapping peaks. See Methods for experimental details.

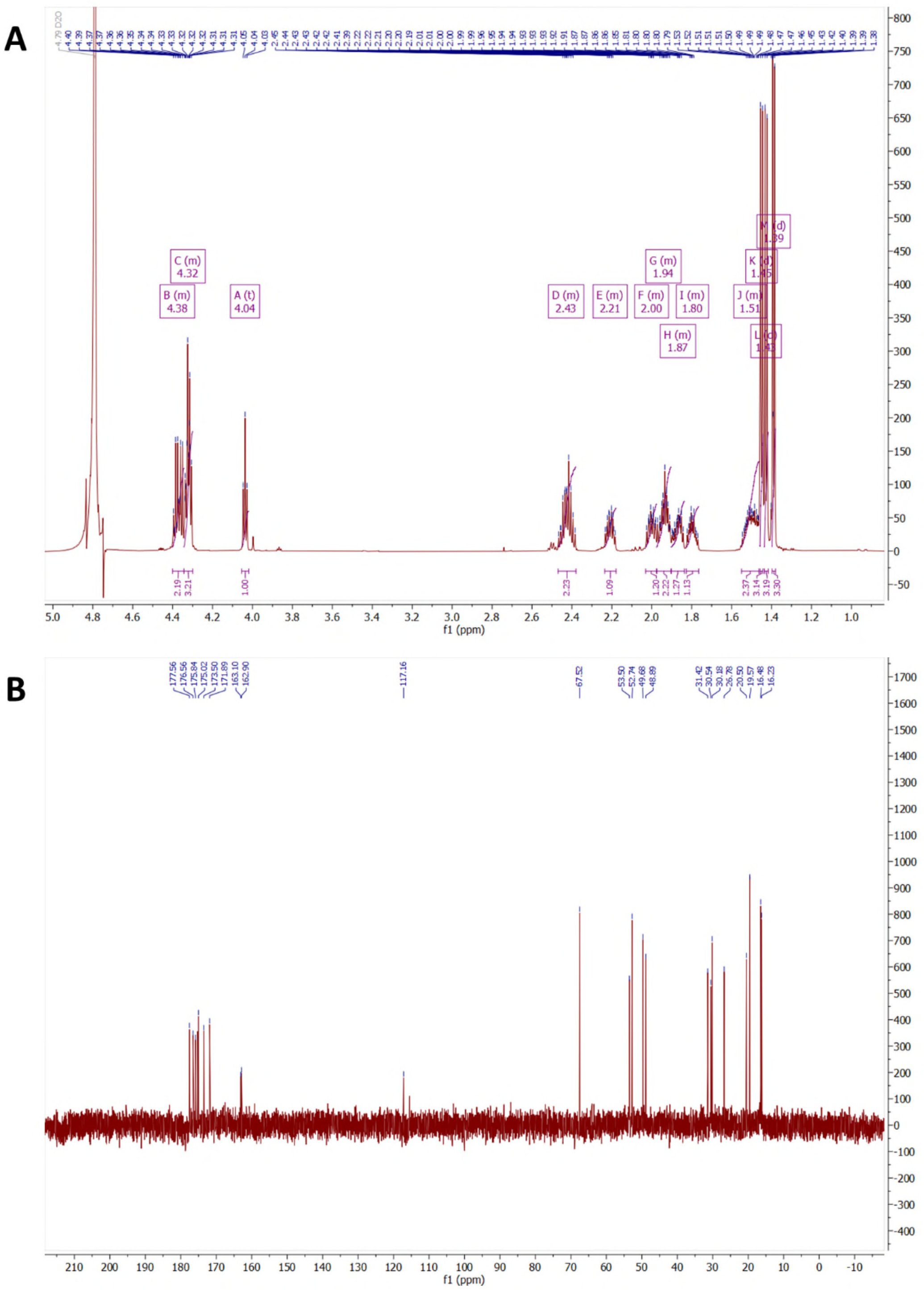

Figure S8. NMR characterisation (700 MHz) of substrate 3 in D_2O. [continues]

C

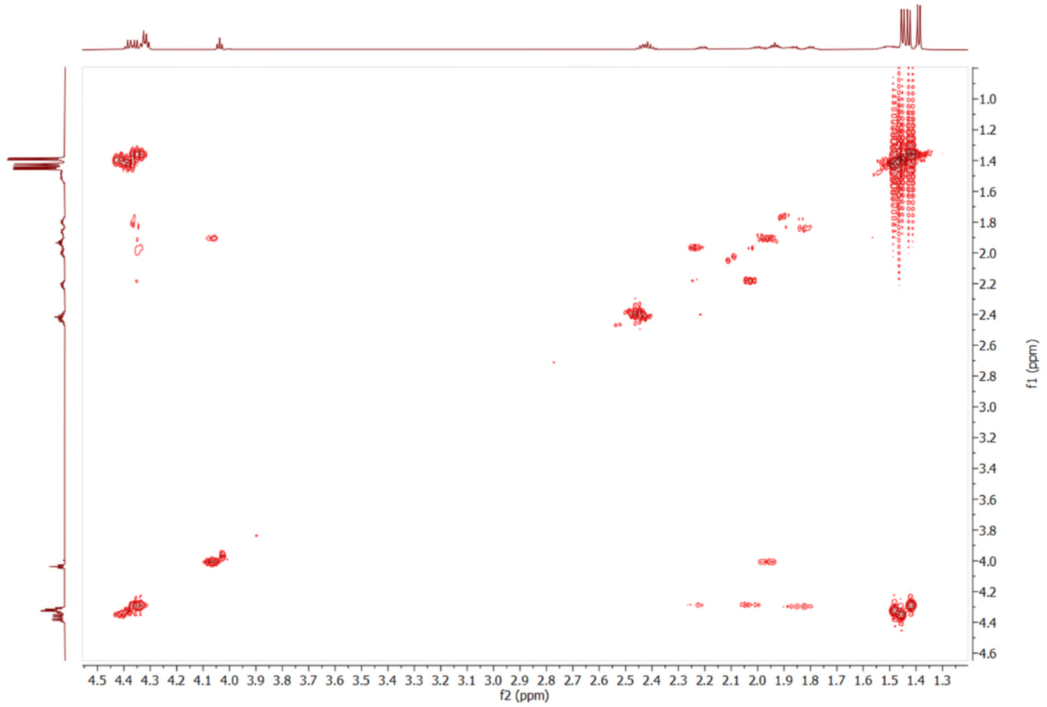

D

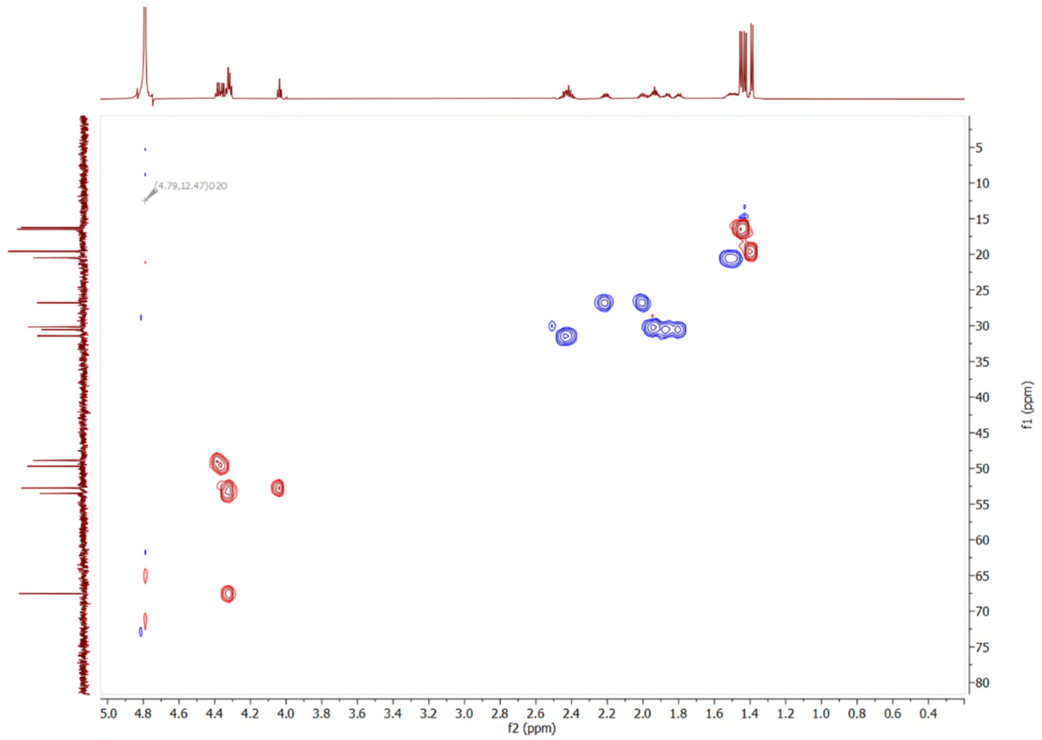

Figure S8. NMR characterisation (700 MHz) of substrate 3 in D_2O. [continues]

E

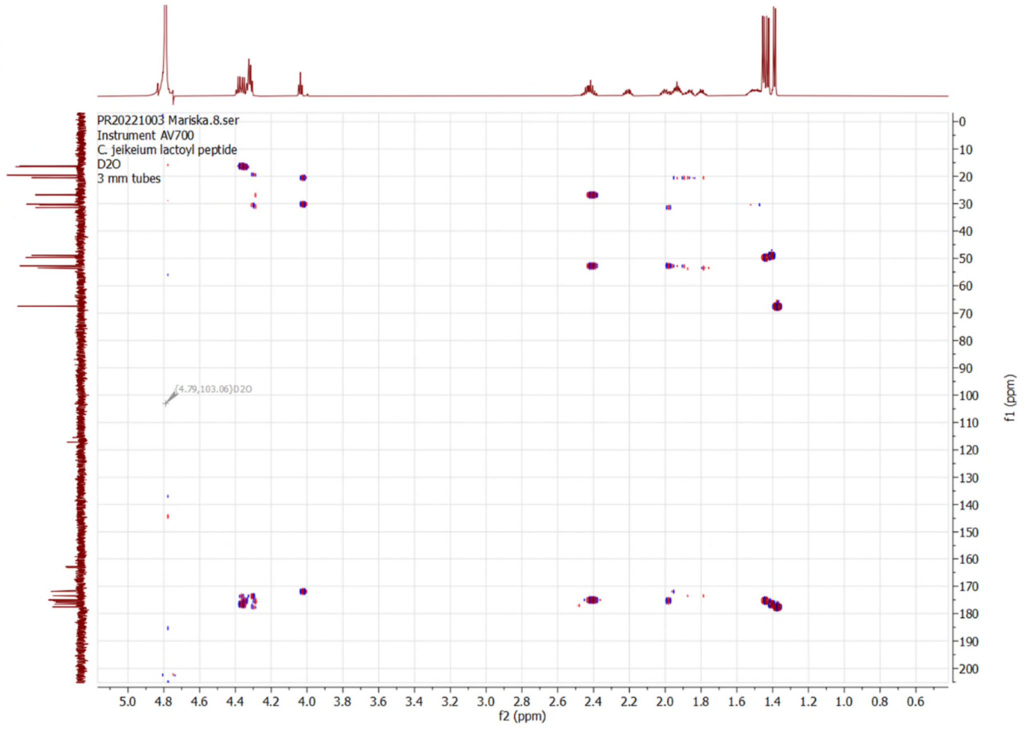

F

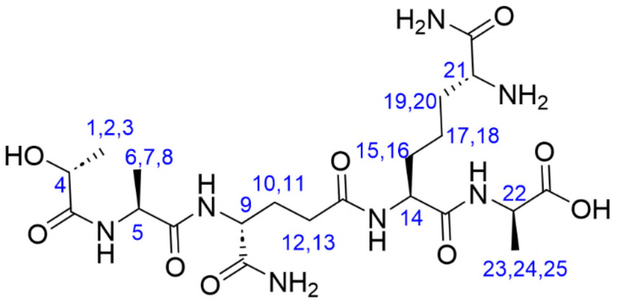

3

Chemical shift (ppm)	Multiplicity	Integration	Assignment
4.40 – 4.35	m	2H	5,22
4.35 – 4.30	m	3H	4,9,14
4.04	t (*J* = 6.6 Hz)	1H	21
2.47 – 2.38	m	2H	10,11
2.23 – 2.18	m	1H	12,13
2.03 – 1.98	m	1H	12,13
1.97 – 1.91	m	2H	19,20
1.90 – 1.84	m	1H	15,16
1.82 – 1.77	m	1H	15,16
1.55 – 1.47	m	2H	17,18
1.45	d (*J* = 7.21 Hz)	3H	6,7,8
1.43	d (*J* = 7.32 Hz)	3H	23,24,25
1.39	d (*J* = 6.91 Hz)	3H	1,2,3

Figure S8. NMR characterisation (700 MHz) of substrate 3 in D_2O. NMR analyses of **3**, obtained from isolation of the cell wall of *C. jeikeium*, comprised of ^1H (**A**), ^{13}C (**B**), ^1H COSY (**C**), ^1H-^{13}C HMBC (**D**) and ^1H-^{13}C HSQC (**E**). **F**. Structure of **3** labelled with assignments. See Methods for experimental details.

A

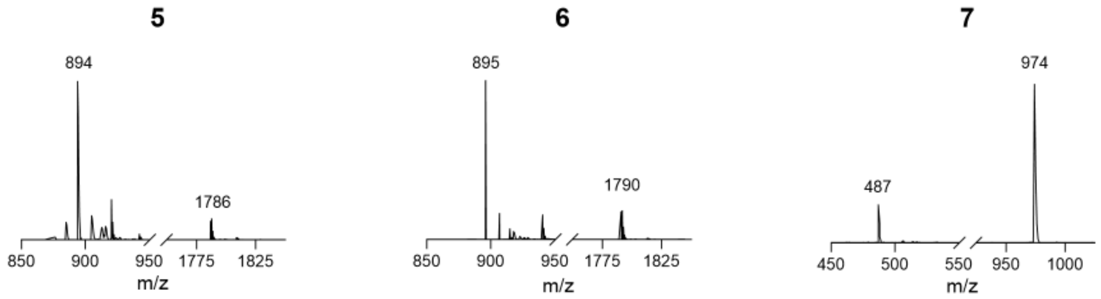

B

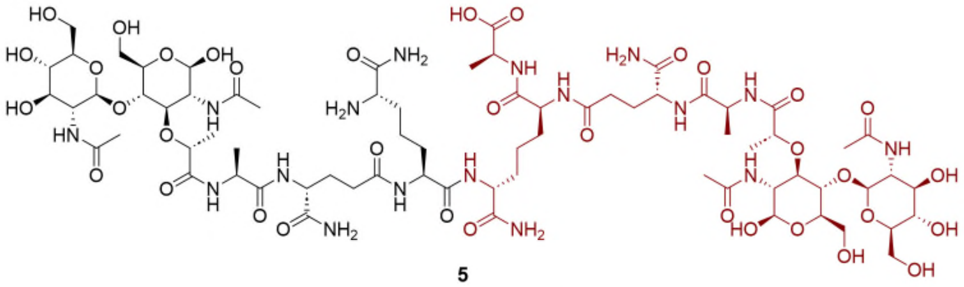

5

Calculated $[M+H]^+$ 1786

Calculated $[M+2H]^{2+}$ 894

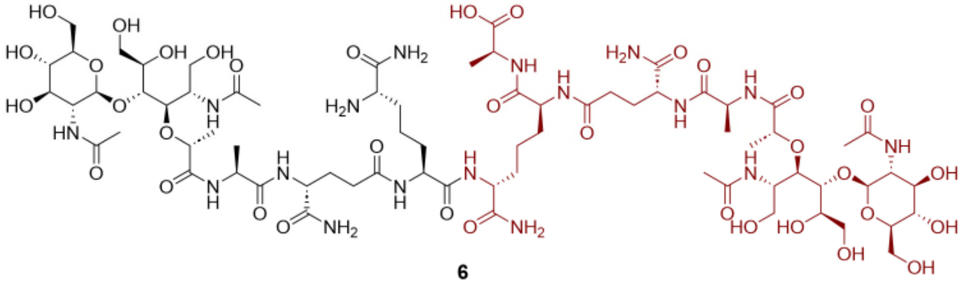

6

Calculated $[M+H]^+$ 1790

Calculated $[M+2H]^{2+}$ 895

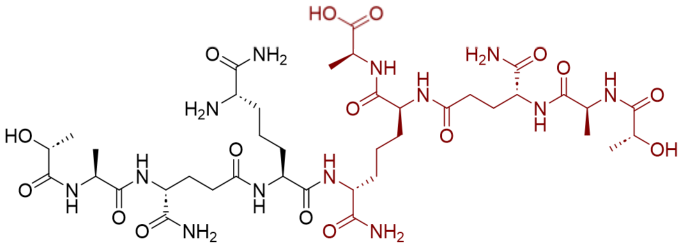

7

Calculated $[M+H]^+$ 974

Calculated $[M+2H]^{2+}$ 487

Figure S9. Mass spectrometric analyses of transpeptidase activity of Ldt$_{Mt2}$. A. MS analysis of Ldt$_{Mt2}$ (5 µM) and **1**, **2** or **3** (100 µM) in 50 mM tris pH 7.5, after 2 hours of incubation at 37 °C show the formation of transpeptidase products **5**, **6**, and **7**, respectively. For **5**, **6** and **7**, both the $[M+H]^+$ and $[M+2H]^{2+}$ masses were observed. See the Methods section for details. Structures of **1**, **2** and **3** are given in Figure 2. **B.** Structures and calculated masses for **5**, **6** and **7**.

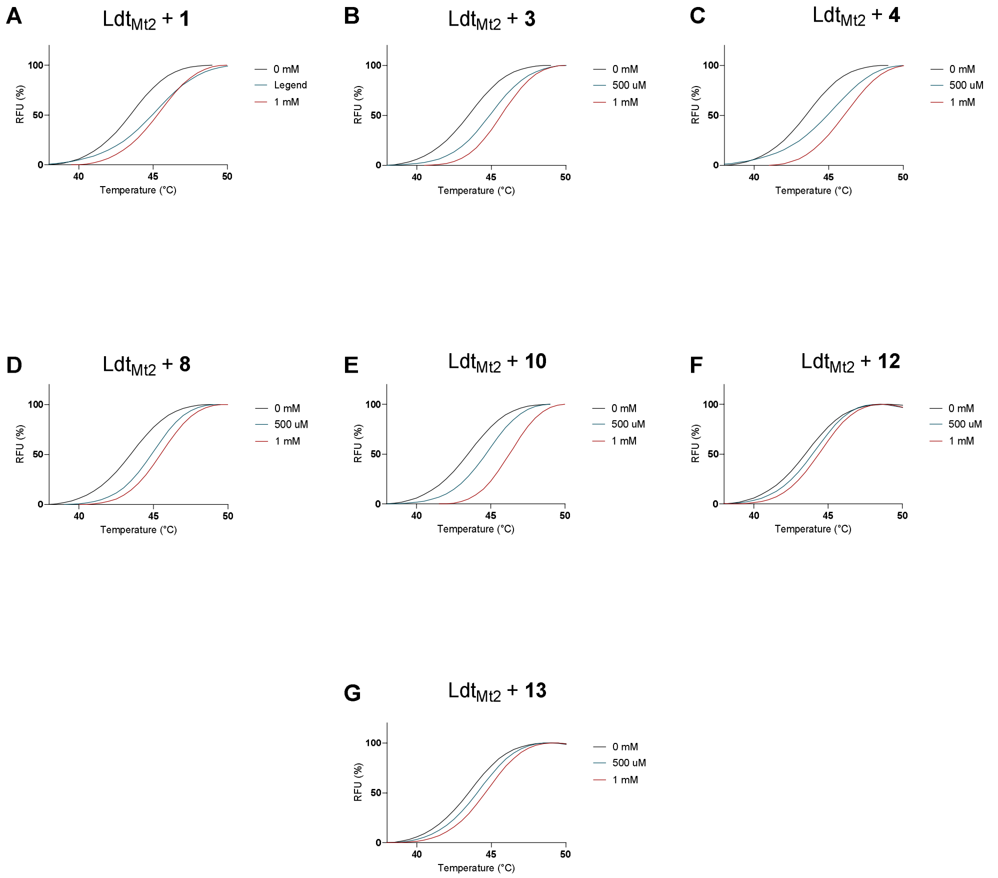

Figure S10. Differential scanning fluorimetry thermal shift curves for Ldt_{Mt2} in the presence of 1, 3, 4, 8, 10, 12, and 13. Ldt_{Mt2} (5 µM) was incubated with peptidoglycan fragments (500 µM or 1 mM) in buffer (50 mM tris pH 7.5) for 30 minutes at room temperature, after which time SYPRO Orange (final concentration 6x, according to manufacturer's definition, Invitrogen) was added. Melting temperatures (T_ms) are provided in Table S2. See Methods for experimental details.

Table S1. Melting temperatures of Ldt_{Mt2} when treated with 1, 3, 4, 8, 10, 12 or 13. The melting temperatures (°C) were obtained in three independent repeats, all of which yielded the same results. Values in parentheses represent the shift in melting temperature compared to unmodified Ldt_{Mt2} (43.5 °C). Values in blue represent a significant change in melting temperature compared to unmodified Ldt_{Mt2} (Δ 2 °C).[4] Structures of **1, 3, 4, 8, 10, 12** and **13** are given in Figure 2.

Compound	T_m 500 μM	T_m 1 mM
**1**	45 (+1.5)	45.5 (+2.0)
**3**	45 (+1.5)	45.5 (+2.0)
**4**	45.5 (+2.0)	46 (+2.5)
**8**	45 (+1.5)	45.5 (+2.0)
**10**	45 (+1.5)	46.5 (+3.0)
**12**	44 (+0.5)	44.5 (+1.0)
**13**	44.5 (+1.0)	45 (+1.5)

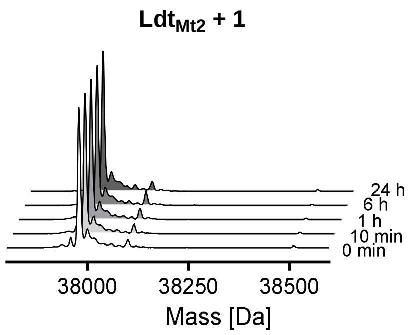

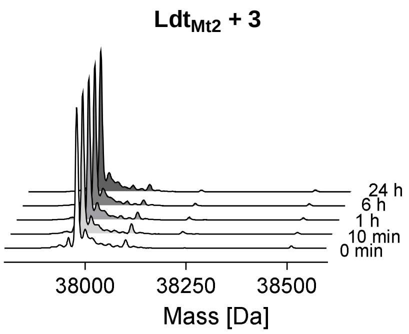

Figure S11. Protein-observed solid-phase extraction mass spectrometric studies of the covalent interaction between Ldt$_{Mt2}$ and tetrapeptides 1 or 3. Ldt$_{Mt2}$ (1 µM) was combined with tetrapeptide **1** or **3** (100 µM), and samples were analysed after the indicated times. Deconvoluted spectra, obtained using the maximum entropy algorithm in the MassHunter Workstation Qualitative Analysis B.07.00 program (Agilent), are shown.

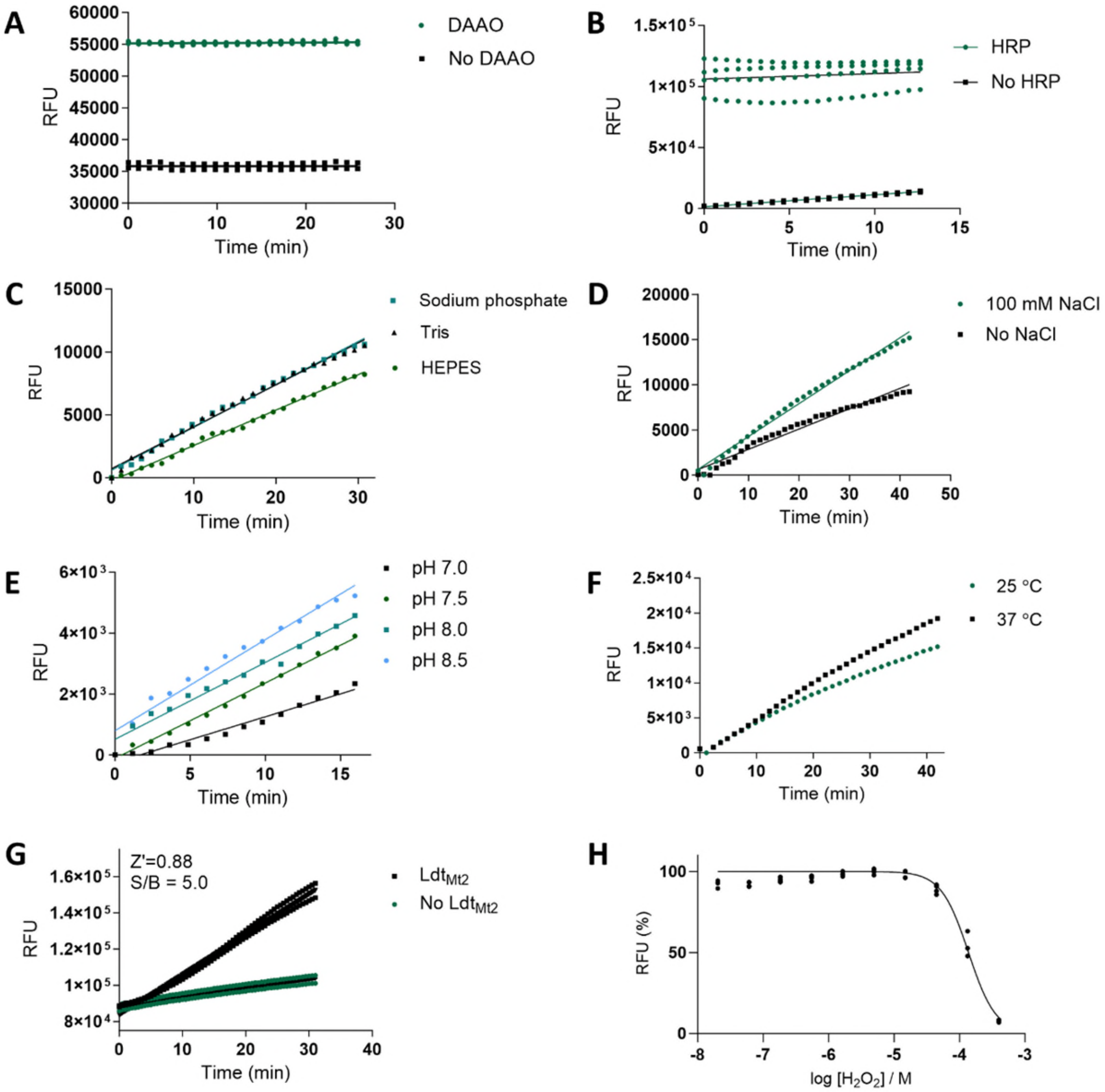

Figure S12. Transpeptidase assay optimisation. A. D-Ala (1 nM) is efficiently oxidised in the presence of D-amino acid oxidase (DAAO; 2 U/mL), horseradish peroxidase (HRP; 2 U/mL), flavin adenine dinucleotide (FAD; 40 µM) and Amplex red (10 µM) in 50 mM sodium phosphate pH 7.5 (n=2). **B**. Hydrogen peroxide was rapidly converted in the presence of HRP (2 U/mL) and Amplex red (10 µM) in 50 mM sodium phosphate pH 7.5 (n=4). **C**. The transpeptidase activity of Ldt_{Mt2} (500 nM) with substrate **1** (35 µM) was assessed in sodium phosphate, tris, and HEPES (50 mM pH 7.5) (n=1). **D**. The transpeptidase activity of Ldt_{Mt2} (500 nM) with substrate **1** (35 µM) was assessed in 50 mM sodium phosphate pH 7.5, with and without the presence of 100 mM NaCl (n=1). **E.** The transpeptidase activity of Ldt_{Mt2} (500 nM) with substrate **1** (35 µM) was assessed in 50 mM sodium phosphate pH with 100 mM NaCl, at pH 7.0-8.5 (n=1). **F**. The transpeptidase activity of Ldt_{Mt2} (500 nM) with substrate **1** (35 µM) was assessed in 50 mM sodium phosphate pH with 100 mM NaCl, pH 8.0 with incubation temperatures of 25 °C or 37 °C (n=1). **G**. The transpeptidase assay of Ldt_{Mt2} (500 nM) with substrate **1** (35 µM) in 50 mM sodium phosphate pH 8.0 with 100 mM NaCl, with incubation at 37 °C (n=3). **H**. Dose-response assay of Ldt_{Mt2} with H_2O_2, using the thiol-reactive fluorogenic probe assay for Ldt_{Mt2} (n=4).[5] Assays in **C**-**G** were performed in the presence of DAAO (2 U/mL), HRP (2 U/mL), FAD (40 µM) and Amplex red (10 µM). See Methods for experimental details. Individual data points are shown.

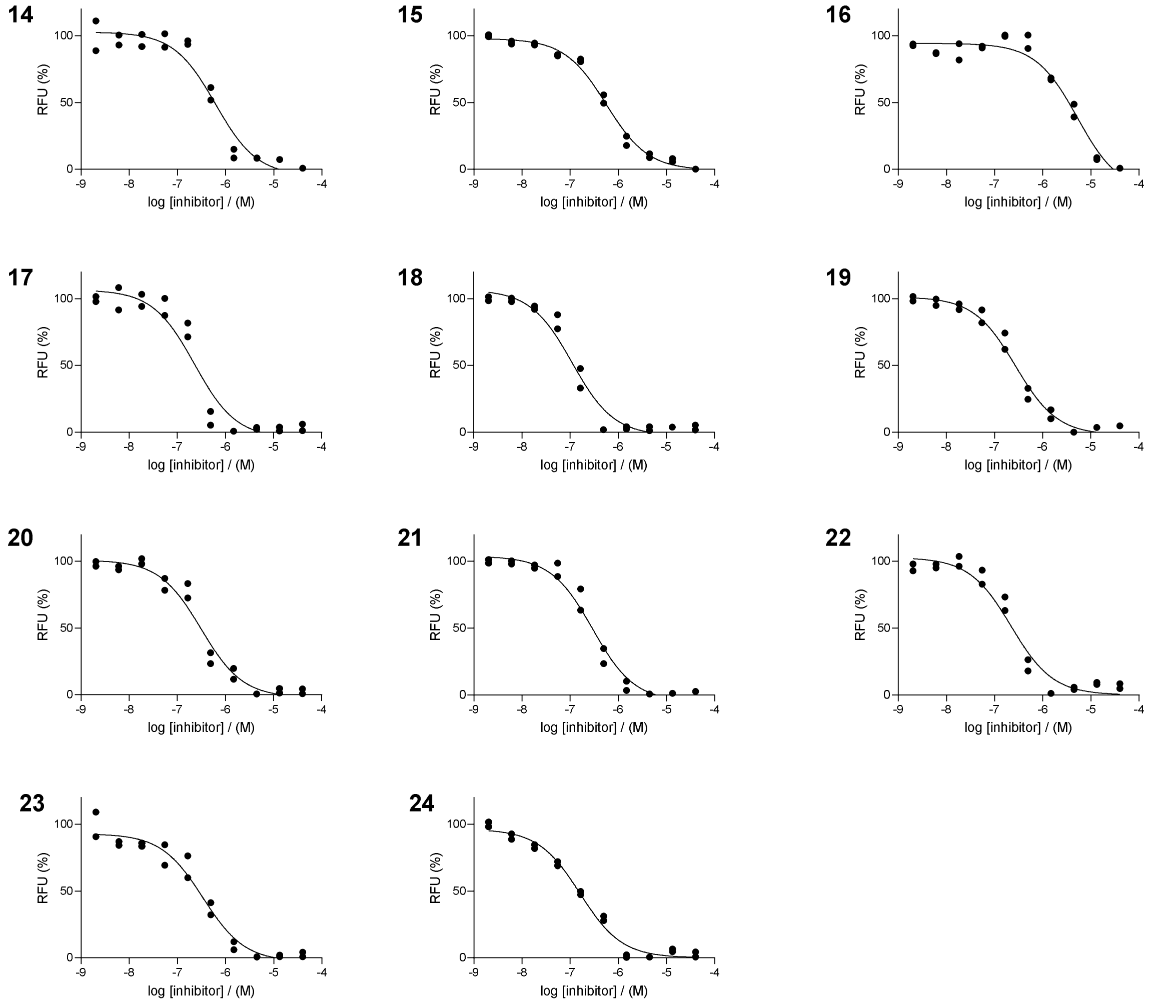

Figure S13. Dose-response curves for inhibition of Ldt_{Mt2} using the transpeptidase assay with compounds 14-24. Ldt_{Mt2} (500 nM) was incubated with the specified inhibitor for 30 minutes at room temperature, after which time the activity was assessed using **1** (35 µM) in the presence of DAAO (2 U/mL), HRP (2 U/mL), FAD (40 µM) and Amplex red (10 µM) in 50 mM sodium phosphate pH 8.0 with 100 mM NaCl at 37 °C. The pIC_{50} values and structures of the compounds are provided in Table S1. See Methods for experimental details. Individual data points are shown.

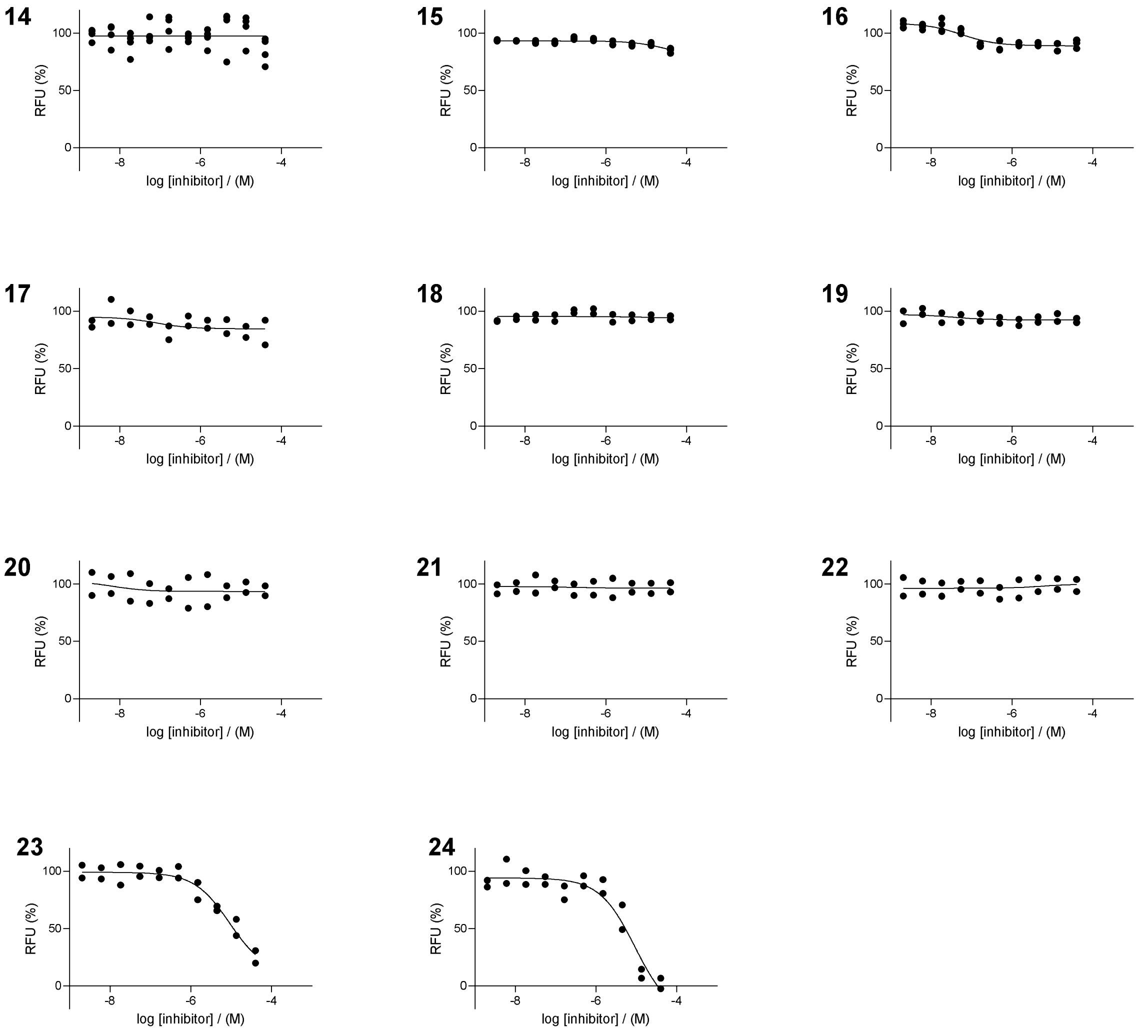

Figure S14. Dose-response curves of the transpeptidase interference assay with compounds 14-24. The specified inhibitors were incubated with DAAO (2 U/mL), HRP (2 U/mL), FAD (40 μM) and Amplex red (10 μM) in 50 mM sodium phosphate pH 8.0 with 100 mM NaCl at room temperature for 30 minutes, after which time D-alanine (500 nM) was added. The pIC_{50} values and compound structures are shown in Table S1. See Methods for experimental details. Individual data points are shown.

Table S2. pIC$_{50}$ values of 14-24 for Ldt$_{Mt2}$ obtained using: (i) the transpeptidase activity assay, (ii) the thiol-reactive fluorescent probe assay,[6,7] and (iii) the transpeptidase interference assay. Assays were performed in quadruplicate. See Methods for experimental details.

Compound	Structure	Transpeptidase activity assay pIC$_{50}$ (mean ± SD)	Fluorogenic probe assay pIC$_{50}$ (mean ± SD)	Transpeptidase interference assay pIC$_{50}$ (mean ± SD)
**14** (Meropenem)	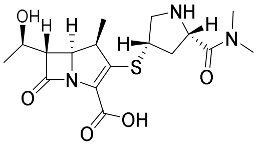	6.2 ± 0.2	5.9 ± 0.04	<4.0
**15** (Faropenem)	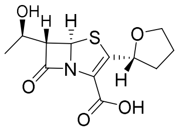	6.3 ± 0.1	6.4 ± 0.03	<4.0
**16**	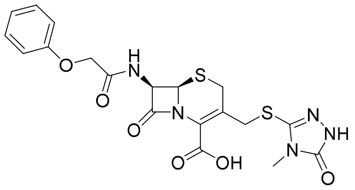	5.3 ± 0.2	5.6 ± 0.1	<4.0
**17**	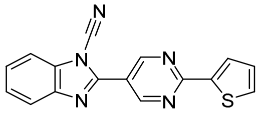	6.6 ± 0.3	7.3 ± 0.06	<4.0
**18**	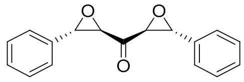	6.9 ± 0.3	7.2 ± 0.1	<4.0
**19**	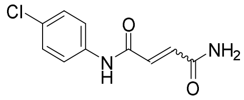	6.6 ± 0.1	6.4 ± 0.08	<4.0
**20**	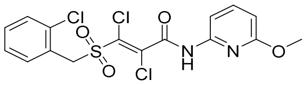	6.5 ± 0.3	7.2 ± 0.04	<4.0
**21**	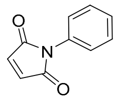	6.5 ± 0.2	7.2 ± 0.03	<4.0
**22**	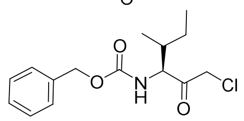	6.7 ± 0.3	7.1 ± 0.01	<4.0
**23**	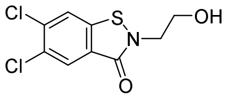	6.5 ± 0.2	8.0 ± 0.04	5.0 ± 0.2
**24** (Ebselen)	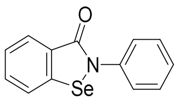	6.8 ± 0.2	6.7 ± 0.04	5.0 ± 0.4

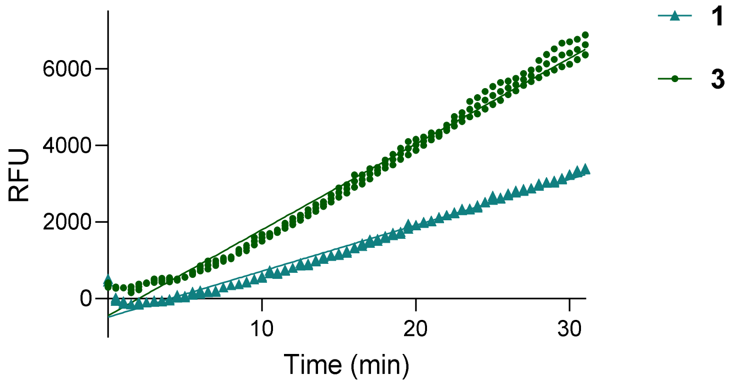

Figure S15. Activity of Ldt_{Mt2} with 1 and 3 assessed using the transpeptidase fluorescence assay. Ldt_{Mt2} (500 nM) activity was assayed using **1** or **3** (35 μM) in the presence of DAAO (2 U/mL), HRP (2 U/mL), FAD (40 μM) and Amplex red (10 μM) in 50 mM sodium phosphate pH 8.0 with 100 mM NaCl at 37 °C (n=3). See Methods for experimental details. Individual data points are shown.

Table S3. Enzyme kinetics of Ldt_{Mt2} with substrates 1 and 3. Ldt_{Mt2} (500 nM) activity was assayed using varying concentrations of **1** or **3** in the presence of DAAO (2 U/mL), HRP (2 U/mL), FAD (40 μM) and Amplex red (10 μM) in 50 mM sodium phosphate pH 8.0 with 100 mM NaCl at 37 °C. See Methods for experimental details. Errors represent standard deviation.

	**K_m (M), x 10^{-3}**	**V_{max} (M min^{-1}), x 10^{-8}**	**k_{cat} (min^{-1}), x 10^{-2}**	**k_{cat}/K_M (min^{-1} M^{-1})**
**1**	Ambiguous	Ambiguous	-	-
**3**	6.2 ± 1.3	1.4 ± 0.5	2.6 ± 0.9	4.2

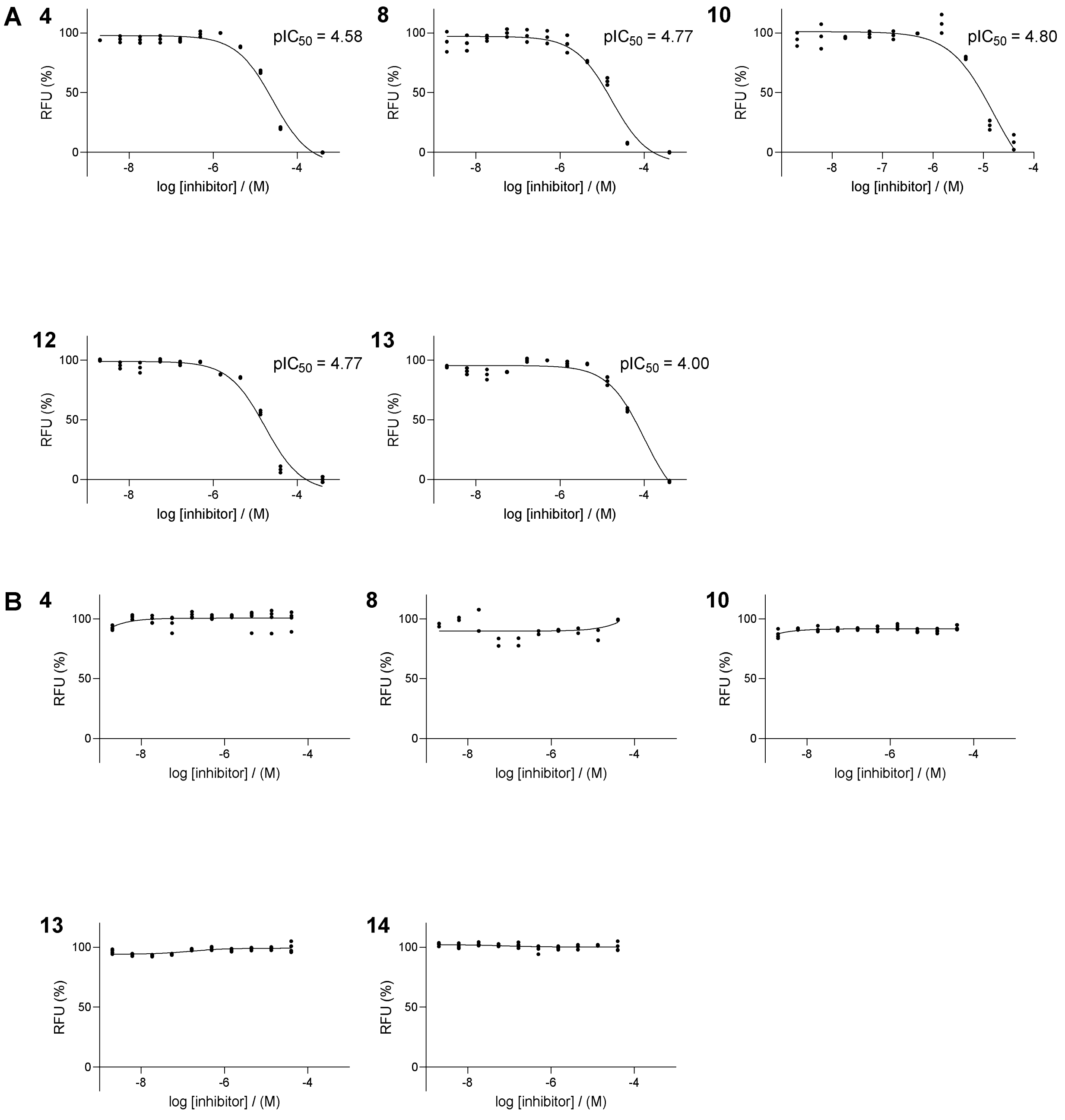

Figure S16. Inhibition studies of Ldt_{Mt2} with non-substrate peptidoglycan fragments. A. Ldt_{Mt2} (500 nM) was incubated with the specified peptidoglycan fragments (**4**, **8**, **10**, **13**, and **14**) for 30 minutes at room temperature, after which time activity was assessed using **1** (35 μM) in the presence of DAAO (2 U/mL), HRP (2 U/mL), FAD (40 μM) and Amplex red (10 μM) in 50 mM sodium phosphate pH 8.0 with 100 mM NaCl at 37 °C (n=3). **B.** Transpeptidase interference assay for peptidoglycan fragments. The specified peptidoglycan fragments (**4**, **8**, **10**, **13**, or **14**) were incubated with DAAO (2 U/mL), HRP (2 U/mL), FAD (40 μM) and Amplex red (10 μM) in 50 mM sodium phosphate pH 8.0 with 100 mM NaCl at room temperature for 30 minutes, after which D-alanine (500 nM) was added (n=3). See Methods for experimental details. Individual data points are shown.

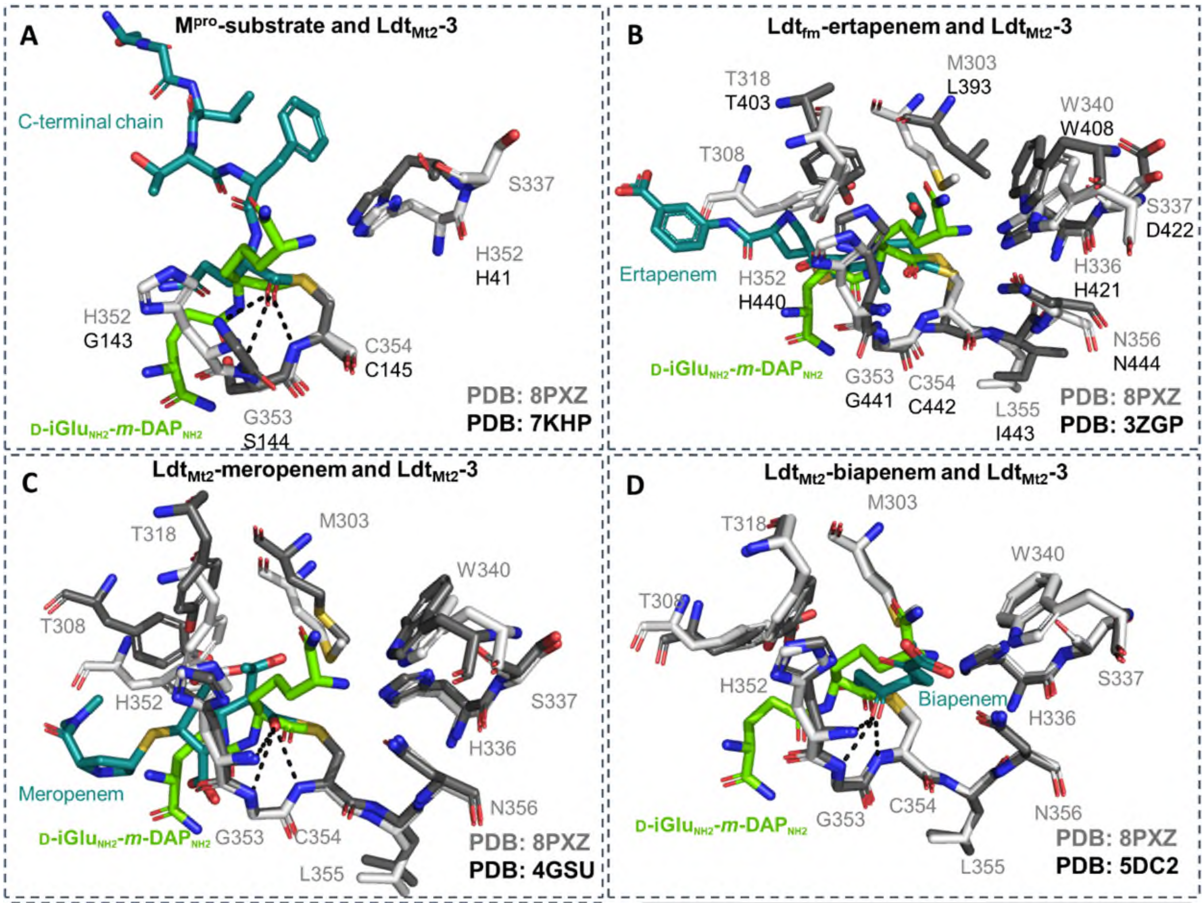

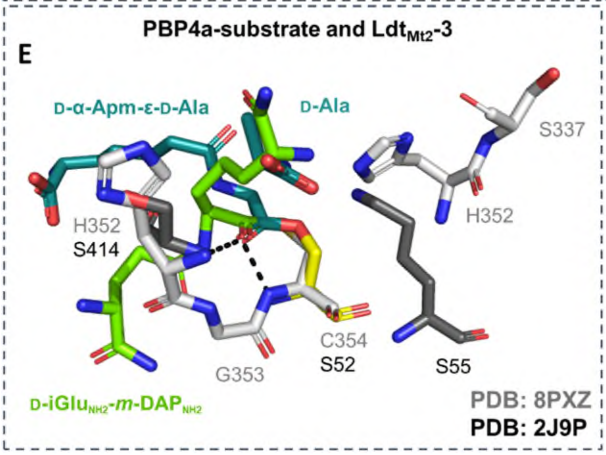

F

Enzyme-acyl complex	Oxyanion residue 1	Oxyanion residue 2	Oxyanion residue 3
Ldt_{Mt2}-3	C354 2.3 Å	G353 3.3 Å	H352 3.8 Å
M^{pro}-substrate	C145 3.0 Å	S144 3.4 Å	G143 3.0 Å
Ldt_{fm}-ertapenem	C442 3.0 Å	G441 3.1 Å	H440 4.5 Å
Ldt_{Mt2}-meropenem	C354 2.9 Å	G353 3.5 Å	H352 3.8 Å
Ldt_{Mt2}-biapenem	C354 2.7 Å	G353 2.7 Å	H352 2.7 Å
PBP4a-substrate mimetic	S52 2.5 Å	S414 2.8 Å	-

Figure S17. Comparison of active site views of different nucleophilic cysteine acyl-enzyme complexes with that of Ldt_{Mt2} in complex with 3. A. View of the oxyanion hole and catalytic residues of the SARS-CoV-2 main protease ($M^{pro)}$ in complex with the C-terminal chain (dark grey and teal; PDB 7KHP)[8] overlaid with one of Ldt_{Mt2} in complex with **3** (light grey and green; PDB 8PXZ). **B**. View of the active site of the *E. faecium* Ldt_{fm} in complex with ertapenem (dark grey and teal; PDB 3ZGP)[9] overlaid with one of Ldt_{Mt2} in complex with **3** (light grey and green; PDB 8PXZ). **C**. View of the active site of Ldt_{Mt2} in complex with meropenem (dark grey and teal; PDB 4GSU)[10] overlaid with one of Ldt_{Mt2} in complex with **3** (light grey and green; PDB 8PXZ). **D.** View of the active site of Ldt_{Mt2} in complex with biapenem (dark grey and teal; PDB 5DC2)[11] overlaid with one of Ldt_{Mt2} in complex with **3** (light grey and green; PDB 8PXZ). **E.** View of the oxyanion hole and catalytic residues of the *B. subtilis* PBP4a in complex with the D-α-aminopimelyl-ε-D-alanine dipeptide (dark grey and green, with Ser52 in yellow; PDB 2J9P)[1] overlaid with one of Ldt_{Mt2} in complex with **3** (light grey and green; PDB 8PXZ). **F**. Distance information defining the position of the acyl group oxygen with respect to the backbone nitrogen atoms of the oxyanion hole residues of the corresponding enzyme. The structure of **3** is given in Figure 2. The structures of biapenem, ertapenem and meropenem are given in Figure S23.

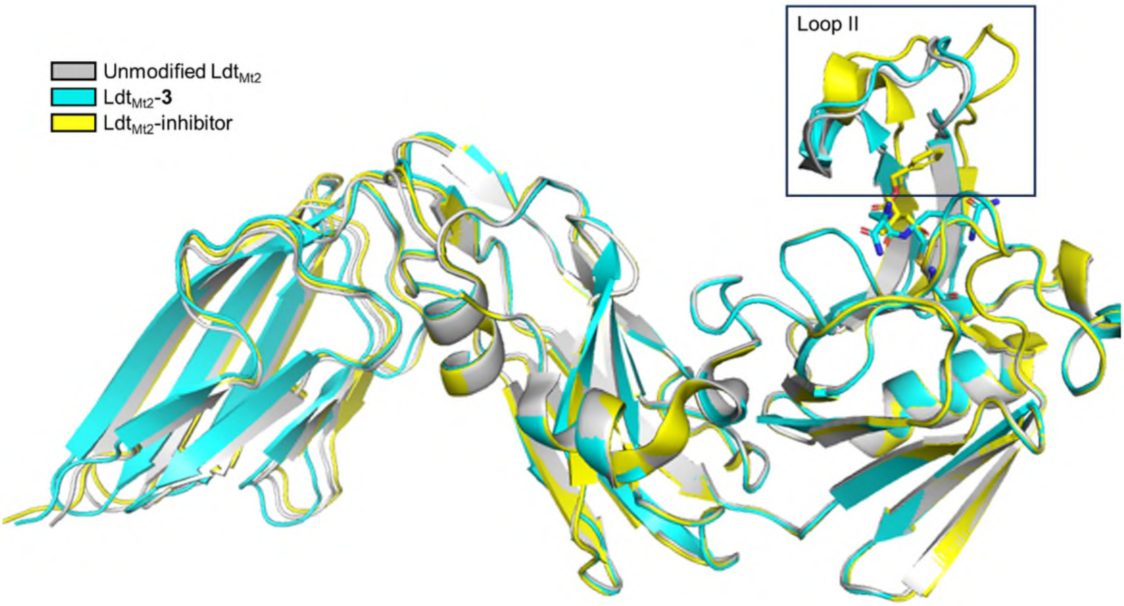

Figure S18. Superimposition of views from structures of unmodified Ldt_{Mt2} (PDB: 6RLG; grey), Ldt_{Mt2} reacted with 3 (PDB: 8PXZ; cyan) and Ldt_{Mt2} reacted with a covalent inhibitor (PDB: 8A1L, yellow). Loop II is observed to undergo conformational changes upon inhibitor binding, as illustrated with a structure of Ldt_{Mt2} reacted with an α-chloroketone inhibitor.[6] However, the overall structure of Ldt_{Mt2} in complex with **3** closely resembles unmodified Ldt_{Mt2}, including with respect to the position of loop II (RMSD 0.65 Å).

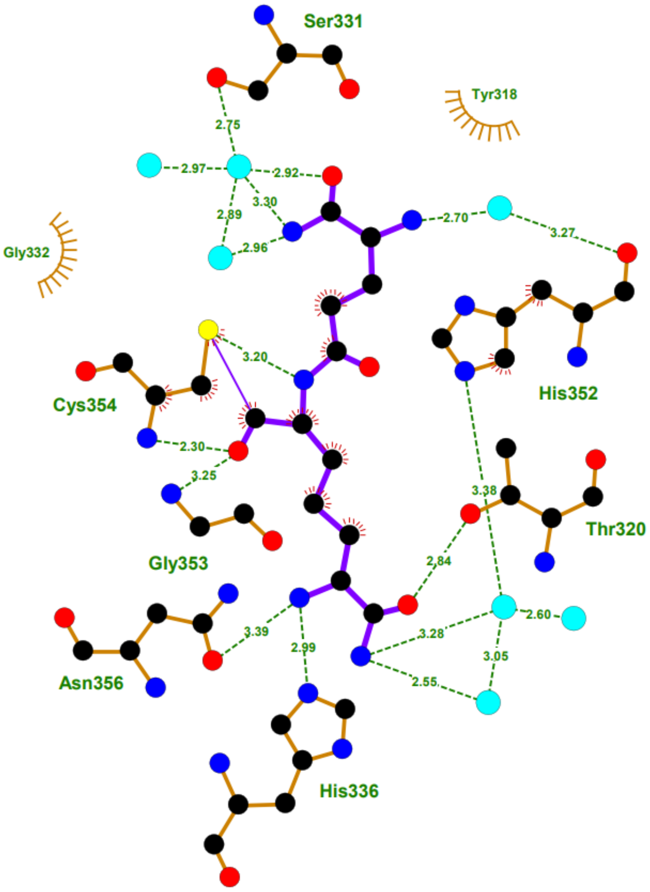

Figure S19. Interactions of the thioester complex of 3 with Ldt$_{Mt2}$. Hydrogen bonds are shown as green dotted lines and covalent bonds are shown as purple lines. **3** is shown in purple. The figure was generated using LigPlot+.[12]

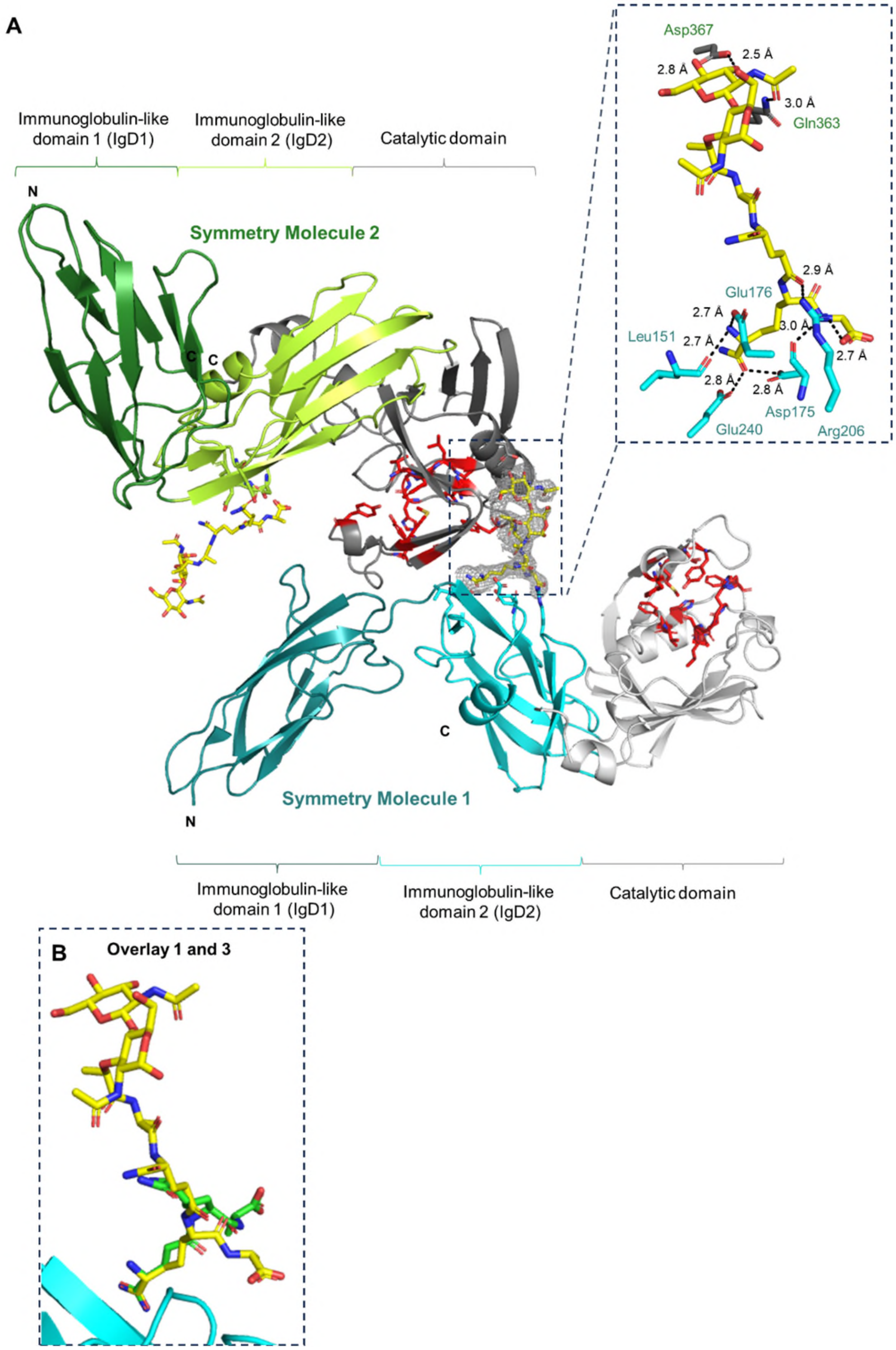

Figure S20. Views from a crystal structure of Ldt$_{Mt2}$^{C354S} in complex with peptidoglycan monomer 1 (yellow, PDB 8PXY) showing interactions with two symmetry related molecules of Ldt$_{Mt2}$^{C354S}. **A.** The $mF_0 - DF_c$ polder OMIT map[13] contoured at 3.0 σ, and carved around **1** is in grey mesh for symmetry molecule 1. Polar interactions are shown in grey dashes. Labels of residues interacting with symmetry molecule 1 are in teal, and labels of residues interacting with symmetry molecule 2 are in green. The active site is in red. The crystallographically assigned structure of **1** is consistent with the structure shown in Figure 2A. **B.** Overlay of the IgD2 bound substrates **1** (yellow, PDB 8PXY), and **3** (green, PDB 8PXZ).

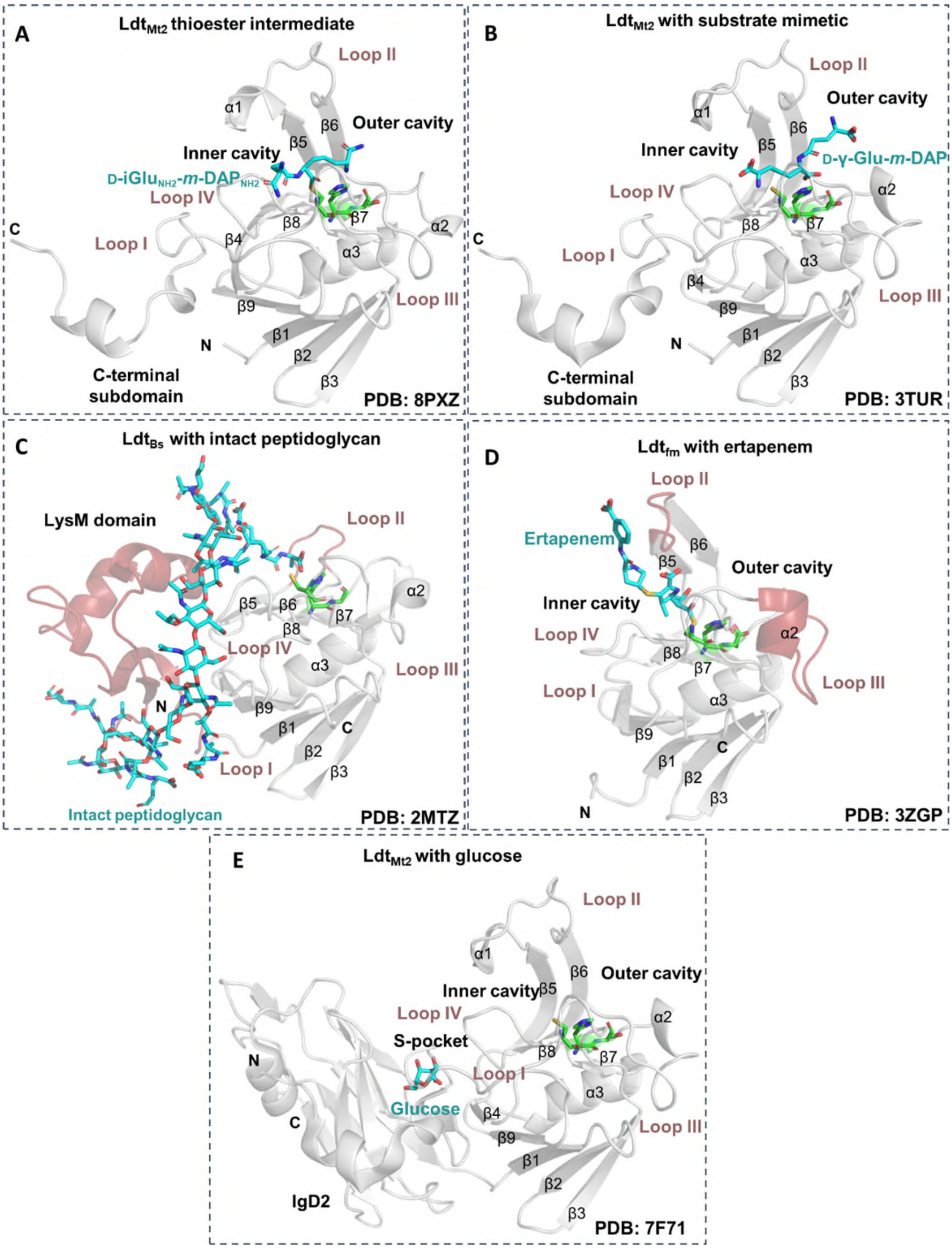

Figure S21. Comparison views of views from structures of L,D-transpeptidases in complex with various peptidoglycan fragments. A. View from our X-ray structure of the catalytic domain of Ldt_{Mt2} in complex with **3** (PDB:8PXZ). **B.** View of the catalytic domain from the X-ray structure of Ldt_{Mt2} in complex with the substrate analogue D-γ-Glu-*m*-DAP (**13**, PDB: 3TUR).[14] **C.** View from a solid-state NMR derived structure of the catalytic domain of Ldt_{Bs} from *Bacillus subtilis* in complex with intact peptidoglycan (PDB: 2MTZ).[15] **D.** View from an NMR structure of the catalytic domain of Ldt_{fm} of *Enterococcus faecium* in complex with ertapenem (PDB: 3ZGP).[9] **E.** View of the catalytic domain and IgD2 from an X-ray crystal structure of Ldt_{Mt2} in complex with glucose (PDB: 7F71).[16] All structural views are oriented following alignment with Ldt_{Mt2}. Proteins are shown in grey, ligands in cyan and catalytic triads in green. Areas in red in **C** and **D** represent significant structural differences with Ldt_{Mt2}. The structures of **3** and **13** are given in Figure 2. The structure of ertapenem is given in Figure S23.

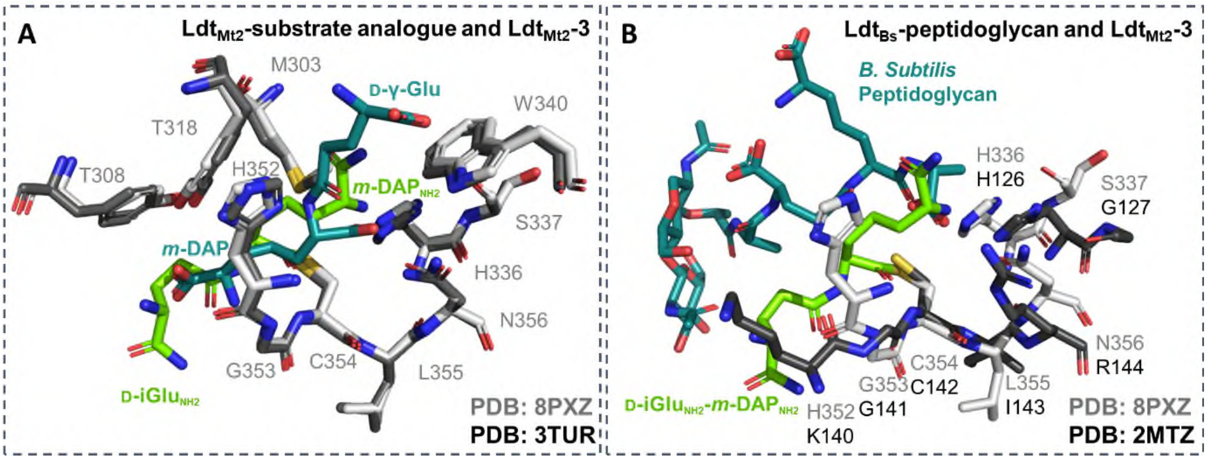

Figure S22. Comparison of the active site of Ldt_{Mt2} in complex with 3 with related structures. A. View of the active site of Ldt_{Mt2} in complex with the substrate analogue D-γ-Glu-*m*-DAP (**13**; dark grey and teal; PDB 3TUR)[14] and Ldt_{Mt2} in complex with **3** (light grey and green; PDB 8PXZ). **B.** View of the active site of the *B. subtilis* Ldt_{Bs} (dark grey) in complex with intact peptidoglycan (shown here is a monomer in teal; PDB: 2MTZ)[15] and Ldt_{Mt2} in complex with **3** (light grey and green; PDB 8PXZ). The structures of **3** and **13** are given in Figure 2.

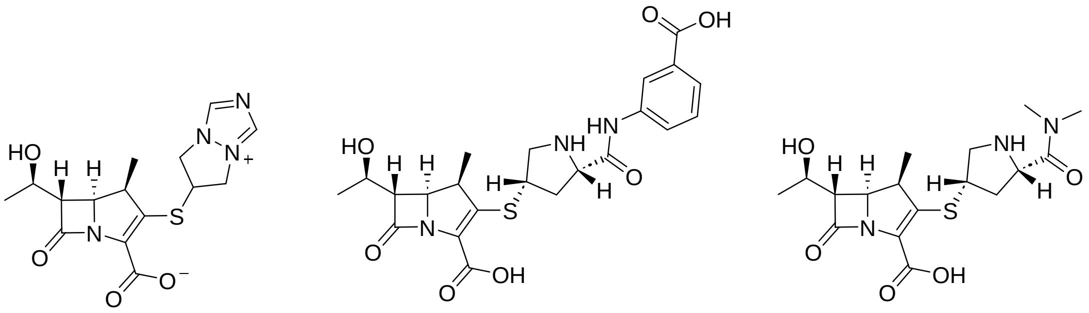

Biapenem Ertapenem Meropenem

Figure S23. Structures of biapenem, ertapenem, and meropenem.

A

B

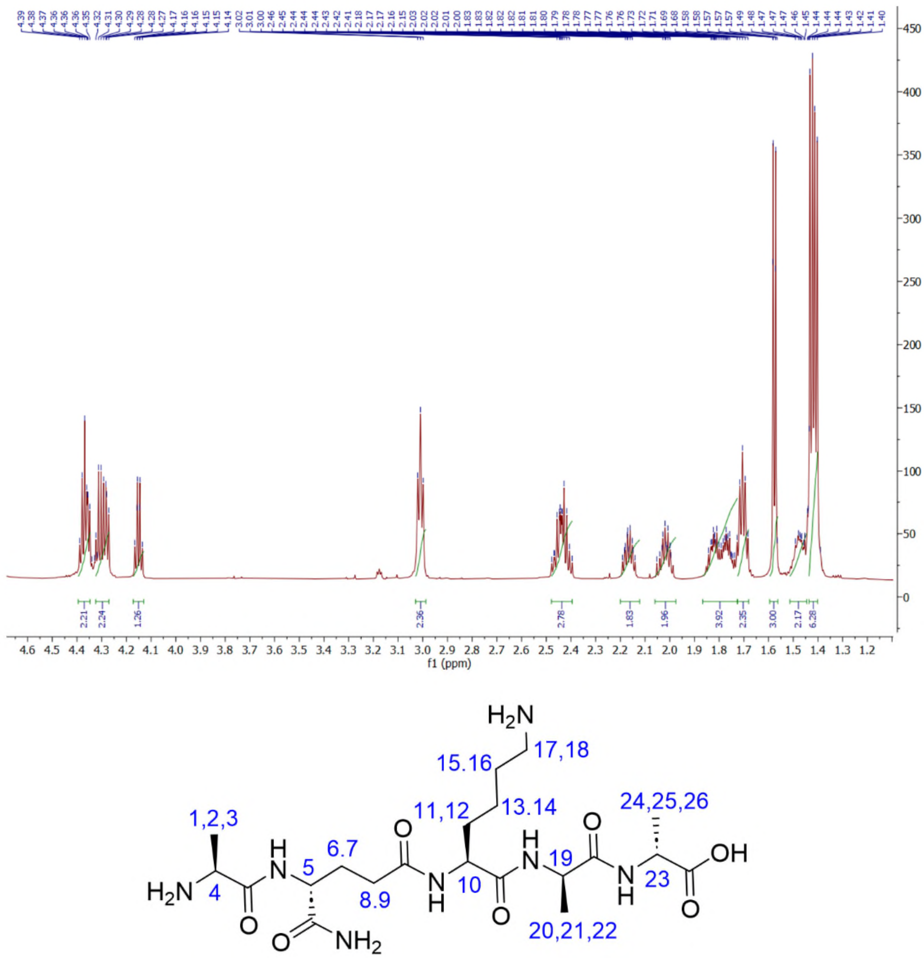

Chemical shift (ppm)	Multiplicity	Integration	Assignment
4.39 – 4.34	m	2H	4,5
4.32 – 4.27	m	2H	10,19
4.17 – 4.13	m	1H	23
3.00	t (*J* = 7.7 Hz)	2H	17,18
2.47 – 2.39	m	2H	Impurity
2.19 – 2.14	m	2H	6,7
2.06 – 1.99	m	2H	8,9
1.85 – 1.73	m	4H	11,12,15,16
1.70	q (*J* = 7.7 Hz)	2H	Impurity
1.58	d (*J* = 7.1 Hz)	3H	1,2,3
1.51 – 1.44	m	2H	13,14
1.42	dd (*J* = 13.6, 7.2 Hz)	6H	20,21,22,24,25,26

Figure S24. Characterisation of peptidoglycan analogue 9 by ^{1}H (700 MHz) NMR in D_2O. A. ^{1}H spectrum of **9**. Note that **9** was prepared by solid-phase peptide synthesis, where D-alanine-Wang resin was coupled with Fmoc protected amino acids, followed by cleavage and deprotection using CF_3CO_2H/CH_2Cl_2 (2:1). **B**. Structure of **9** labelled with assignments. See Methods for experimental details.

A

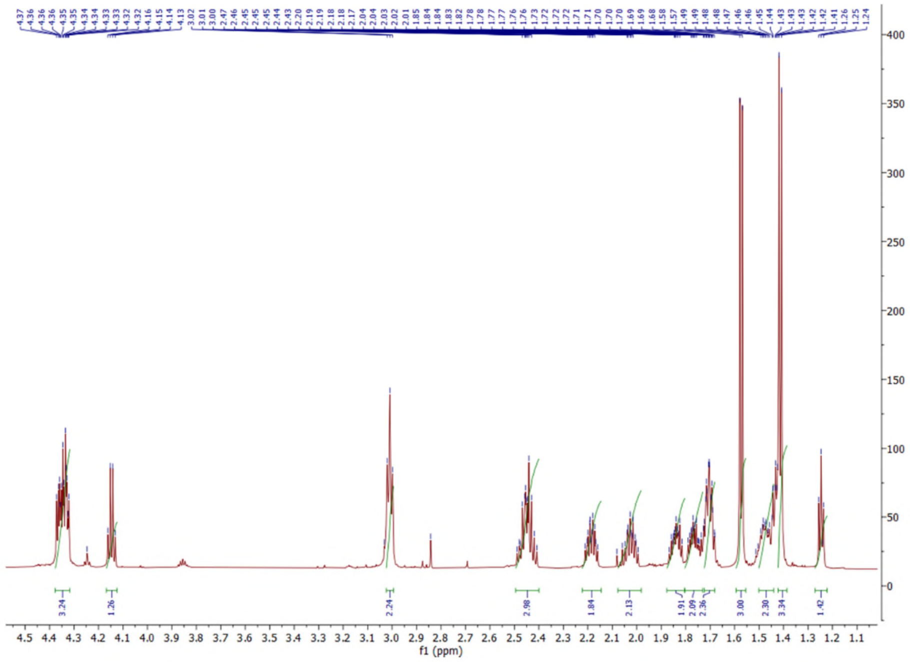

B

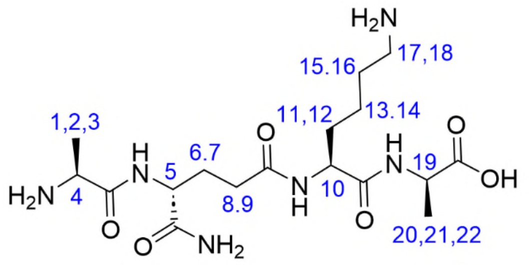

Chemical shift (ppm)	Multiplicity	Integration	Assignment
4.35-4.32	m	3H	4, 5,10
4.15	q (*J* = 7.1 Hz)	1H	19
3.00	t (*J* = 7.7 Hz)	2H	17,18
2.49-2.41	m	3H	Impurity
2.21-2.15	m	2H	6,7
2.08-1.99	m	2H	8,9
1.87-1.81	m	2H	11,12
1.79-1.74	m	2H	15,16
1.73-1.68	m	2H	Impurity
1.57	d (*J* = 7.1 Hz)	3H	1,2,3
1.50-1.44	m	2H	13,14
1.41	d (*J* = 7.3 Hz)	3H	20,21,22
1.25	t (*J* = 6.8 Hz)	1H	Impurity

Figure S25. Characterisation of peptidoglycan analogue 11 by ^{1}H (700 MHz) NMR in D_2O. A. ^{1}H spectrum of **11**. Note that **11** was prepared by solid-phase peptide synthesis, using D-alanine-Wang resin and coupling with Fmoc protected amino acids, followed by cleavage and deprotection using CF_3CO_2H/CH_2Cl_2 (2:1). **B**. Structure of **11** labelled with assignments. See Methods for experimental details.

References

1 Sauvage, E., Duez, C., Herman, R., Kerff, F., Petrella, S., Anderson, J. W., Adediran, S. A., Pratt, R. F., Frère, J.-M. & Charlier, P. Crystal Structure of the *Bacillus subtilis* Penicillin-binding Protein 4a, and its Complex with a Peptidoglycan Mimetic Peptide. *J. Mol. Biol.* **371**, 528-539 (2007).

2 Sauvage, E., Kerff, F., Terrak, M., Ayala, J. A. & Charlier, P. The penicillin-binding proteins: structure and role in peptidoglycan biosynthesis. *FEMS Microbiol. Rev.* **32**, 234-258 (2008).

3 de Munnik, M., Lohans, C. T., Lang, P. A., Langley, G. W., Malla, T. R., Tumber, A., Schofield, C. J. & Brem, J. Targeting the *Mycobacterium tuberculosis* transpeptidase Ldt_{Mt2} with cysteine-reactive inhibitors including ebselen. *Chem. Commun.* **55**, 10214-10217 (2019).

4 Huynh, K. & Partch, C. L. Analysis of Protein Stability and Ligand Interactions by Thermal Shift Assay. *Curr. Protoc. Protein Sci.* **79**, 14-21 (2015).

5 de Munnik, M., Lohans, C. T., Langley, G. W., Bon, C., Brem, J. & Schofield, C. J. A Fluorescence-Based Assay for Screening β-Lactams Targeting the *Mycobacterium tuberculosis* Transpeptidase Ldt_{Mt2}. *ChemBioChem* **21**, 368-372 (2020).

6 de Munnik, M., Lang, P. A., De Dios Antos, F., Cacho, M., Bates, R. H., Brem, J., Rodríguez-Miquel, B. & Schofield, C. J. High-Throughput Screen with the L,D-transpeptidase Ldt_{Mt2} of *Mycobacterium tuberculosis* Reveals Novel Classes of Covalently Reacting Inhibitors *Chem. Sci.* **14**, 7262-7278 (2023).

7 de Munnik, M., Lithgow, J., Brewitz, L., Christensen, K. E., Bates, R. H., Rodriguez-Miquel, B. & Schofield, C. J. αβ,α'β'-Diepoxyketones are mechanism-based inhibitors of nucleophilic cysteine enzymes. *Chem. Commun. (Camb)* **59**, 12859-12862 (2023).

8 Lee, J., Worrall, L. J., Vuckovic, M., Rosell, F. I., Gentile, F., Ton, A.-T., Caveney, N. A., Ban, F., Cherkasov, A., Paetzel, M. & Strynadka, N. C. J. Crystallographic structure of wild-type SARS-CoV-2 main protease acyl-enzyme intermediate with physiological C-terminal autoprocessing site. *Nat. Commun.* **11** (2020).

9 Lecoq, L., Dubée, V., Triboulet, S., Bougault, C., Hugonnet, J.-E., Arthur, M. & Simorre, J.-P. Structure of *Enterococcus faecium* L,D-Transpeptidase Acylated by Ertapenem Provides Insight into the Inactivation Mechanism. *ACS Chem. Biol.* **8**, 1140-1146 (2013).

10 Kim, H. S., Kim, J., Im, H. N., Yoon, J. Y., An, D. R., Yoon, H. J., Kim, J. Y., Min, H. K., Kim, S.-J. & Lee, J. Y. Structural basis for the inhibition of *Mycobacterium tuberculosis* L,D-transpeptidase by meropenem, a drug effective against extensively drug-resistant strains. *Acta Crystallogr. D* **69**, 420-431 (2013).

11 Bianchet, M. A., Pan, Y. H., Basta, L. A. B., Saavedra, H., Lloyd, E. P., Kumar, P., Mattoo, R., Townsend, C. A. & Lamichhane, G. Structural insight into the inactivation of *Mycobacterium tuberculosis* non-classical transpeptidase Ldt_{Mt2} by biapenem and tebipenem. *BMC Biochem.* **18** (2017).

12 Laskowski, R. A. & Swindells, M. B. LigPlot+: Multiple Ligand–Protein Interaction Diagrams for Drug Discovery. *J. Chem. Inf. Model.* **51**, 2778-2786 (2011).

13 Liebschner, D., Afonine, P. V., Moriarty, N. W., Poon, B. K., Sobolev, O. V., Terwilliger, T. C. & Adams, P. D. Polder maps: improving OMIT maps by excluding bulk solvent. *Acta Crystallogr. D* **73**, 148-157 (2017).

14 Erdemli, S. B., Gupta, R., Bishai, W. R., Lamichhane, G., Amzel, L. M. & Bianchet, M. A. Targeting the cell wall of *Mycobacterium tuberculosis*: structure and mechanism of L,D-transpeptidase 2. *Structure* **20**, 2103-2115 (2012).

15 Schanda, P., Triboulet, S., Laguri, C., Bougault, C. M., Ayala, I., Callon, M., Arthur, M. & Simorre, J.-P. Atomic Model of a Cell-Wall Cross-Linking Enzyme in Complex with an Intact Bacterial Peptidoglycan. *J. Am. Chem. Soc.* **136**, 17852-17860 (2014).

16 Ahmad, N., Dugad, S., Chauhan, V., Ahmed, S., Sharma, K., Kachhap, S., Zaidi, R., Bishai, W. R., Lamichhane, G. & Kumar, P. Allosteric cooperation in β-lactam binding to a non-classical transpeptidase. *eLife* **11**, e73055 (2022).
