# Supplementary material for: Biochemical and crystallographic studies of l,d-transpeptidase 2 from Mycobacterium tuberculosis with its natural monomer substrate
Source: Commun Biol. 2024 Sep 18;7:1173. doi: 10.1038/s42003-024-06785-3 (PMC11410929; doi:10.1038/s42003-024-06785-3)
Supplement: Supplementary file 2 — Description of Additional Supplementary Materials [file 42003_2024_6785_MOESM2_ESM.pdf]

Description of Additional Supplementary Files

File name: Supplementary Data 1

Description: all source data
